# The GhWL1‐GhH1‐GhGA2OX1 Transcriptional Module Regulates Cotton Leaf Morphology

**DOI:** 10.1002/advs.202410783

**Published:** 2025-04-30

**Authors:** Jingjing Zhan, Xiaoshuang Zhang, Ye Wang, Hang Zhao, Yu Chu, Peng Wang, Yanli Chen, Xi Wei, Wenqiang Qin, Menghan Liu, Jie Kong, Fuguang Li, Xiaoyang Ge

**Affiliations:** ^1^ State Key Laboratory of Cotton Bio‐breeding and Integrated Utilization Institute of Cotton Research of Chinese Academy of Agricultural Sciences Anyang 455000 China; ^2^ College of Plant Science and Technology of Huazhong Agricultural University Wuhan 430000 China; ^3^ College of Life Sciences Qufu Normal University Qufu 273100 China; ^4^ Institute of Economic Crops Xinjiang Academy of Agricultural Sciences Urumqi Xinjiang 830091 China

**Keywords:** cotton, GhH1, GhWL1, leaf morphology, T‐DNA mutant

## Abstract

Leaf morphology critically influences photosynthetic efficiency, directly affecting crop yield and quality. In this study, a T‐DNA insertion mutant (*wl‐D*), characterized by a wrinkled‐leaf phenotype, is identified. Genetic analysis reveals that this phenotype is governed by a single dominant gene, *WRINKLED‐LEAF 1 (GhWL1)*, which is highly expressed in *wl‐D* compared to the wild type (WT). Overexpression of *GhWL1* in WT caused curling at leaf edges, while suppression of *GhWL1* in *wl‐D* restored normal leaf morphology, validating its functional role. Further analysis demonstrated that *GhWL1* interacts with *GhH1*, a protein with a KNOX1 structural domain, to regulate leaf development. Overexpression of *GhH1* in WT results in a leaf shrinkage phenotype similar to *wl‐D*, whereas suppressing *GhH1* in *wl‐D* restored normal leaf morphology, indicating that *GhH1* acts downstream of *GhWL1*. The GhWL1‐GhH1 complex directly binds to the promoter of *GhGA2OX1* (gibberellin 2‐beta‐dioxygenase 1), positively regulating its expression. Overexpression of *GhGA2OX1* in WT mimicked the leaf shrinkage phenotype observed in plants overexpressing *GhH1*. These findings establish the GhWL1‐GhH1*‐GhGA2OX1* module as a critical pathway in regulating leaf development, offering valuable insights into the genetic and hormonal networks controlling leaf morphological diversity.

## Graphical abstract

This study identified GhWL1 as a key gene involved in regulating leaf development through analysis of a T‐DNA insertion mutant. The GhWL1‐GhH1 complex is shown to positively regulate GhGA2OX1 by directly binding to its promoter, thereby establishing the GhWL1‐GhH1‐GhGA2OX1 module as a critical pathway in leaf development. This finding underscores the module's pivotal role in shaping leaf morphology.

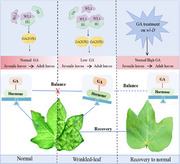

## 1 Introduction

Leaf morphology plays a central role in photosynthesis, transpiration, and respiration, directly influencing plant growth, reproduction, and environmental adaptation.^[^ ^1^, ^2^, ^3^, ^4^, ^5^, ^6^ ^]^ Proper leaf morphology is essential for maximizing photosynthetic efficiency and optimizing crop yield. A variety of leaf development mutants have been identified in species like *Arabidopsis thaliana*, *Zea mays* (maize), *Oryza sativa* (rice), and *Lycopersicon esculentum* (tomato),^[^ ^7^, ^8^ ^]^ offering valuable resources for investigating the genetic frameworks governing leaf development. However, the regulatory network of leaf morphology remains unclear and warrants further investigation.

The molecular mechanisms that govern leaf development involve intricate interactions among multiple genes and signaling pathways. Specifically, transcription factors play pivotal roles throughout the process of leaf morphogenesis.^[^ ^3^ ^]^ For example, Class II TCP transcription factors are widely recognized as key regulators of leaf development, exerting significant influence over leaf size and morphology.^[^ ^9^ ^]^ Suppression of *CIN* genes in *Antirrhinum majus*, *TCP3* and *TCP4* in *Arabidopsis*, and *TCP24* and *TCP29* in tomato have all been associated with abnormal leaf morphology.^[^ ^10^, ^11^, ^12^, ^13^ ^]^ The *KNOX* gene family plays a significant role in various plant developmental processes, particularly leaf development.^[^ ^14^ ^]^ Evidence from leaf dissection indicates that *KNOX* gene expression extends beyond the shoot apical meristem to the leaf primordia, suggesting their involvement in leaf formation.^[^ ^15^ ^]^ In *Arabidopsis*, there are four *KNOX1* genes, namely *SHOOT MERISTEMLESS* (*STM*), *BREVIPEDICELLUS* (*BP*), *KNAT2*, and *KNAT6*. Overexpression of the *STM* gene in *Arabidopsis* results in lobed leaves.^[^ ^16^ ^]^ Similarly, overexpression of the *KNOX1* genes in tomatoes, lettuce, and strawberries leads to the production of compound or deeply serrated leaves.^[^ ^17^, ^18^, ^19^, ^20^ ^]^ Despite this, limited research has been conducted on the role of *KNOX1* genes in cotton, a key crop with significant economic value.

Beyond regulatory genes, the integration of multiple hormones, both at the signaling and hormonal levels, allows for precise regulation of leaf development.^[^ ^21^, ^22^ ^]^ Auxin is essential for orchestrating various facets of leaf development, influencing cellular division, expansion, and differentiation, which are critical for determining leaf size, shape, and patterning.^[^ ^23^ ^]^ Multiple studies have demonstrated that mutations in auxin biosynthesis‐associated genes can lead to alterations in leaf shape.^[^ ^24^, ^25^ ^]^ Gibberellins (GAs) regulate cell proliferation and expansion, and disruptions in genes associated with GA signaling or homeostasis can affect plant organ development.^[^ ^26^ ^]^ For instance, overexpression of *GIBBERELLIN 20‐OXIDASE 1* (*GA20OX1*), a key enzyme involved in GA biosynthesis, increased levels of active GA in *Arabidopsis*, producing larger leaves with increased cell size and number.^[^ ^21^ ^]^ Conversely, the *procera (pro)* mutant exhibits fewer leaflets and smooth margins due to altered GA signaling. Other hormones, including jasmonic acid (JA), abscisic acid (ABA), ethylene, and strigolactones, also contribute to leaf development.^[^ ^27^ ^]^ Understanding the interplay between these hormones and their genetic regulators remains a critical area of research.

Several leaf‐shape mutations in cotton have provided key insights into the genetic and molecular mechanisms underlying leaf development. Turcotte and Feaster^[^ ^28^ ^]^ identified a mutant in “Pima cotton” with shrunken leaves and dark green foliage, controlled by a dominant gene pair labeled as *Ru*. In 1980, further research uncovered another mutant exhibiting progressive leaf shrinkage: leaves remained normal up to the sixth node, became slightly reduced between the seventh and ninth nodes, significantly shrank at the tenth node, and disappeared by the 25th node.^[^ ^29^ ^]^ Another study in 1986 documented a shriveled‐leaf mutant in “Pima cotton,” with visible shriveling starting at the eighth node, controlled by a pair of recessive genes.^[^ ^30^ ^]^ Despite these advancements, the precise genetic drivers of leaf development in cotton remain insufficiently characterized. This study aims to unravel the genetic and molecular mechanisms regulating cotton leaf morphology by examining a gain‐of‐function mutant displaying a wrinkled‐leaf phenotype. This phenotype is associated with altered expression of GA‐related genes, particularly *GhGA2OX1*, orchestrated by the interaction of *GhWL1* and *GhH1*. Our findings reveal a regulatory role for the *GhWL1‐GhH1* module in modulating GA signaling pathways, establishing a direct connection between these interactions and leaf morphological diversity. These results address a long‐standing question of how plants integrate internal signaling pathways to adapt leaf morphology to environmental conditions. By clarifying these mechanisms, this study contributes to a deeper understanding of the genetic networks influencing leaf development and highlights opportunities for manipulating leaf traits to enhance crop resilience and productivity.

## 2 Results

### 2.1 Phenotypic Characterization of the *wl‐D* Mutant

**Figure 1:**
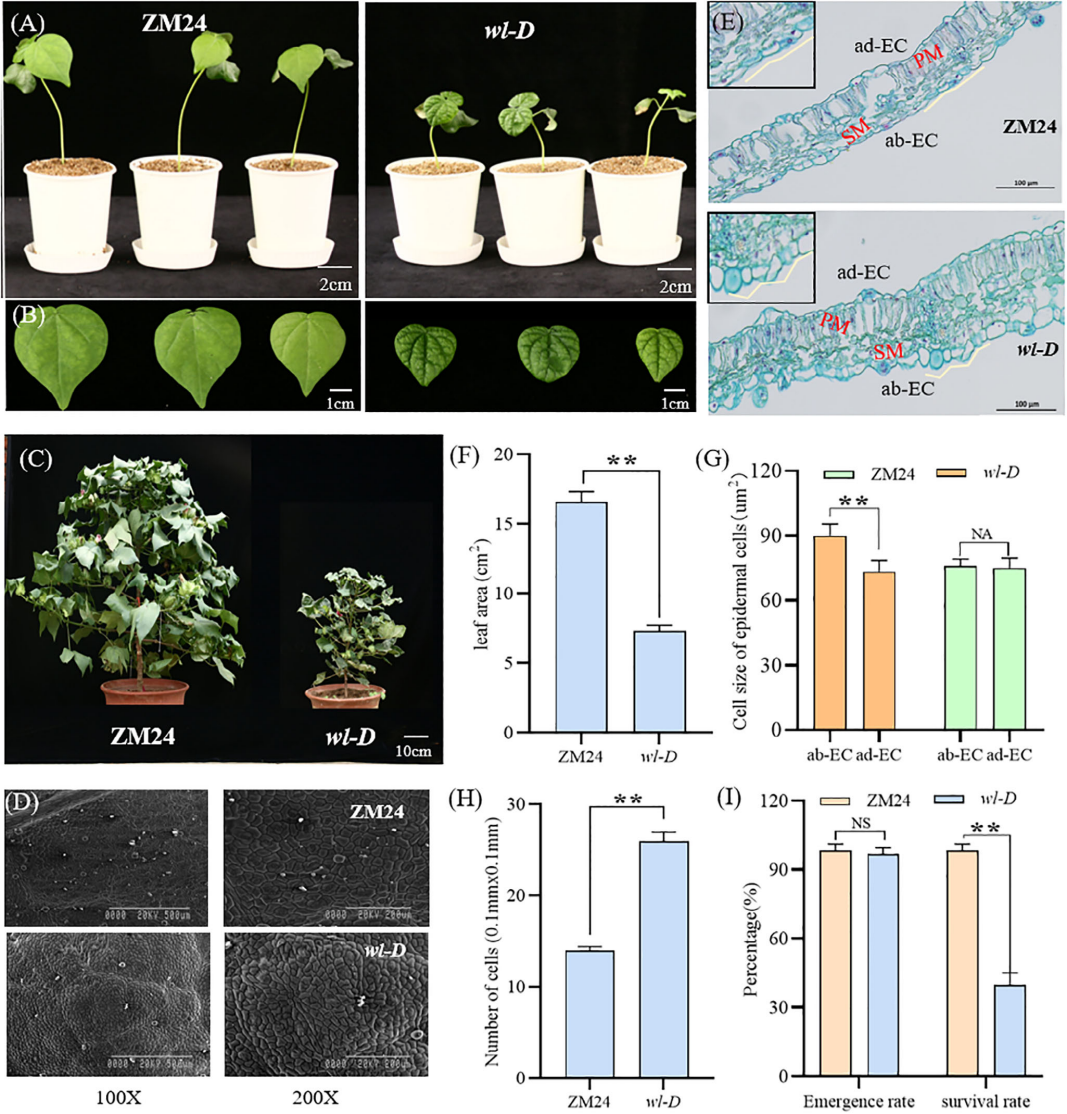
Comparative morphological analysis of the mutant *wl‐D* and ZM24. A,B) Leaf morphology at the early developmental stage displays distinct differences between the mutant *wl‐D* (left) and wild‐type ZM24 (right). C) Comparative plant architecture shows the mutant *wl‐D* (right) and ZM24 (left). D) Scanning electron microscopy (SEM) analysis highlights structural variations in the leaf margins of *wl‐D* (bottom) and ZM24 (top). E) Toluidine blue‐stained paraffin sections reveal histological differences between *wl‐D* and ZM24, indicating variations in adaxial epidermal cells (ad‐EC), abaxial epidermal cells (ab‐EC), palisade mesophyll cells (PM), and spongy mesophyll cells (SM). F) Leaf surface area of 20‐day‐old plants demonstrates significant differences between *wl‐D* and ZM24. G) Measurement of epidermal cell size in (E) indicates a reduction in *wl‐D*. Data are presented as mean ± SD from ten independent experiments (n > 20). H) The number of cells per unit area (0.1 mm × 0.1 mm) differs significantly between *wl‐D* and ZM24 in (D). Data represent mean ± SD for three independent experiments (n > 10). **P < 0.01 (Student's *t*‐test). I) Statistical analysis of seed germination and survival rates reveals no significant difference (NS) but highlights distinct patterns in growth outcomes. **P < 0.01 (Student's *t*‐test).

In this study, we identified the wl‐D mutant from a T‐DNA insertion mutant population in the ZM24 background. This mutant is characterized by a wrinkled‐leaf phenotype, semi‐dwarf stature, and reduced organ size during both vegetative and reproductive stages. The wrinkled‐leaf trait persists throughout the growth cycle. Comparative phenotypic analyses revealed that wl‐D leaves are greener, exhibit a wrinkled surface, and develop yellow spots during specific growth periods, distinguishing them from ZM24 leaves (**Figure** **1**A‐C; Figures S1 and S2, Supporting Information).

Scanning electron microscopy (SEM) of *wl‐D* leaves revealed abnormal epidermal cells and reduced cell size compared to the normal morphology of ZM24 leaves (Figure 1D). Quantitative analyses demonstrated that *wl‐D* leaves have a projected area ≈50% smaller than ZM24 leaves. Moreover, the epidermal cells in *wl‐D* leaves are densely packed, leading to a higher cell density per unit area (Figure 1F,H). Further examination of the abaxial and adaxial epidermis through paraffin sections showed that *wl‐D* plants possess significantly enlarged cells at the abaxial leaf margins compared to ZM24 (Figure 1E,G). While the germination rate of *wl‐D* remains unaffected, its survival rate is significantly lower (Figure 1I). Collectively, these findings suggest that the wrinkled‐leaf phenotype in *wl‐D* arises from irregular cellular arrangement in the abaxial epidermis during leaf development.

### 2.2 Identification of the T‐DNA Insertion Site in *wl‐D*

**Figure 2:**
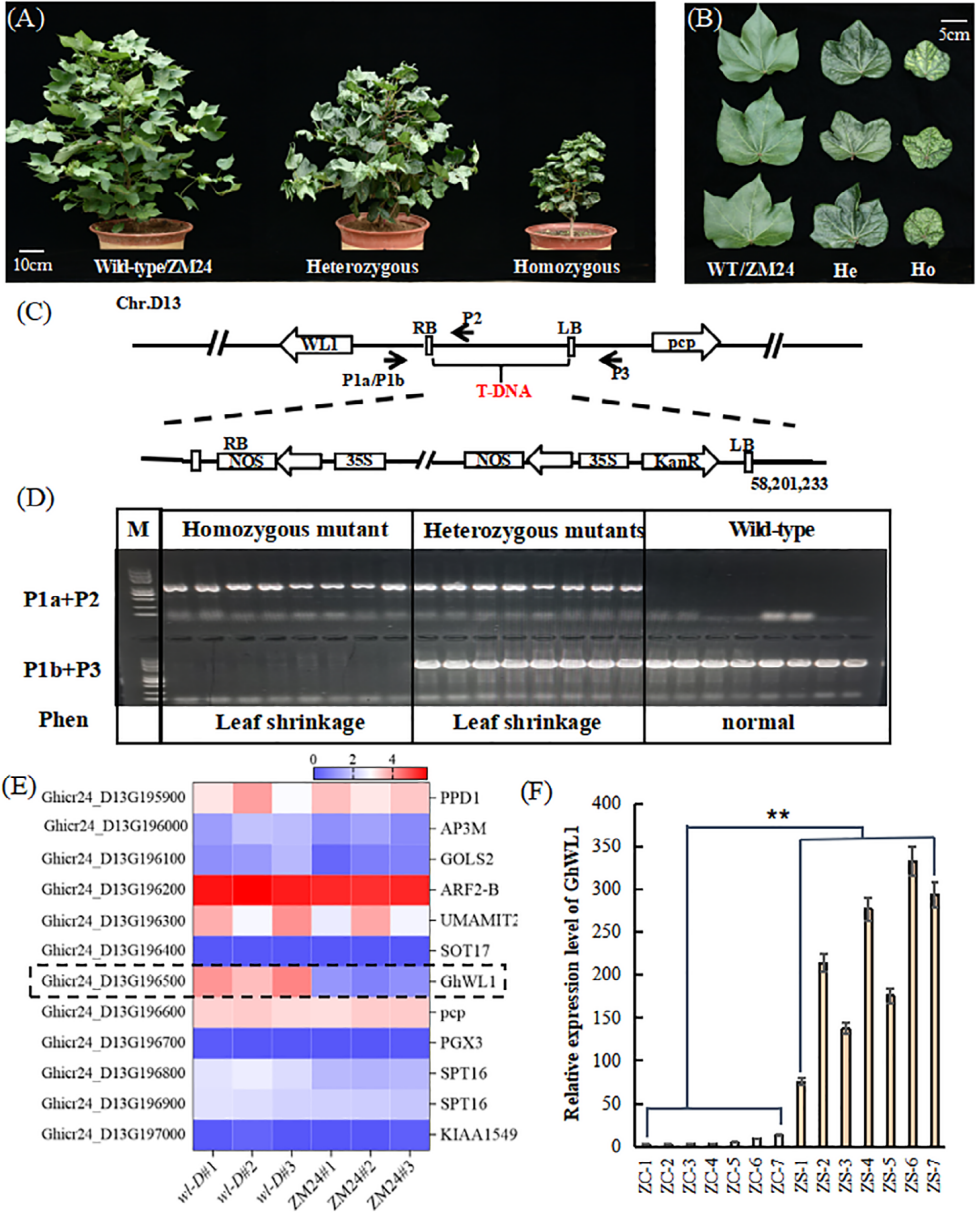
Phenotypic characterization and co‐segregation analysis in the F_2_ population. A,B) Phenotypic segregation within the F_2_ progeny includes wild‐type (WT), heterozygous (He), and homozygous (Ho) categories, with scale bars of 10 cm and 5 cm in (A) and (B), respectively. C) Schematic of T‐DNA insertion site demonstrates primer utilization (P2, P1, P3) for co‐segregation analysis. The diagram includes T‐DNA left border (LB), right border (RB), and the kanamycin resistance gene (Kan). D) Co‐segregation PCR analysis confirms the association of T‐DNA insertion with specific phenotypes in the F_2_ population. Primers P1a (T‐DNA LB) and P2 (gene‐specific), alongside P1b (T‐DNA LB) and P3 (T‐DNA RB), were used for validation. E) RNA‐Seq heatmap illustrates gene expression differences near the T‐DNA site between *wl‐D* and ZM24, with log2‐transformed values indicating expression intensity (blue: low, red: high). Data are derived from three biological replicates. F) RT‐qPCR analysis quantifies *GhWL1* expression in the F_2_ population, using endogenous Histone (His) as the reference. ZC represents plants with normal leaves, while ZS indicates plants with wrinkled leaves. Data are presented as mean ± SD for three independent experiments. **P < 0.01 (Student's *t*‐test).

To investigate whether the phenotypic traits of *wl‐D* are linked to the T‐DNA insertion, a cross was performed between *wl‐D* and ZM24, producing F1 and F2 populations. The F1 plants displayed the wrinkled‐leaf phenotype, identical to *wl‐D* (**Figure** **2**A,B). In the F2 population, the segregation pattern of wrinkled and normal phenotypes followed a Mendelian 3:1 ratio, confirmed by chi‐square analysis (χ^2^ = 0.98, *P* > 0.050, df = 1) (**Table** **1**), indicating single‐gene inheritance.

**Table 1:** Segregation ratio of leaf shrinkage in the F_2_ population.

Phenotype	Number	χ2
Normal Leaf	64	
Leaf Shrinkage	166	
Total	230	0.98

To assess the association between T‐DNA insertion and the wrinkled‐leaf phenotype, co‐segregation analysis was conducted using 40 F_2_ individuals, evenly divided between wrinkled‐leaf and normal phenotypes. Kanamycin resistance, located on the T‐DNA, was used as a marker.^[^ ^31^ ^]^ Leaf segments from the 20 normal plants turned yellow under kanamycin selection, whereas those from the wrinkled‐leaf group remained green (Figure S3A,B, Supporting Information). These results suggest a linkage between T‐DNA insertion and the mutant phenotype.

Thermal asymmetric interlaced PCR (TAIL‐PCR) was employed to identify the precise T‐DNA integration site (Figure S4A, Supporting Information). Sequencing analysis revealed that the T‐DNA was inserted into a non‐coding region on chromosome D13. Genotyping of 164 F_2_ individuals using primers specific to the T‐DNA flanking sequences confirmed that all wrinkled‐leaf individuals harbored the insertion, whereas it was absent in normal‐leaf individuals (Figure 2D; Figure S5, Supporting Information). These findings affirm a genetic linkage between the T‐DNA insertion and the *wl‐D* phenotype.

A BLAST search on the Cotton Genome Annotation Project website identified several genes near the T‐DNA insertion site (Figure 2C; Figure S4B, Supporting Information). Among these, only *Ghicr24_D13G196500* (*GhWL1*) exhibited significant up‐regulation in *wl‐D* compared to ZM24 (Figure 2E; Figure S6, Supporting Information). To further confirm this, RT‐qPCR analysis was conducted on 20‐day‐old seedlings. The results showed markedly higher expression of *GhWL1* in wrinkled‐leaf mutants than in normal‐leaf plants (Figure 2F). These findings demonstrate that the wrinkled‐leaf phenotype in *wl‐D* is co‐segregated with elevated *GhWL1* expression, implicating the T‐DNA insertion near the *Ghicr24_D13G196500* locus as the driver of the observed phenotype.

### 2.3 GhWL1 Functional Analysis in Leaf Development

**Figure 3:**
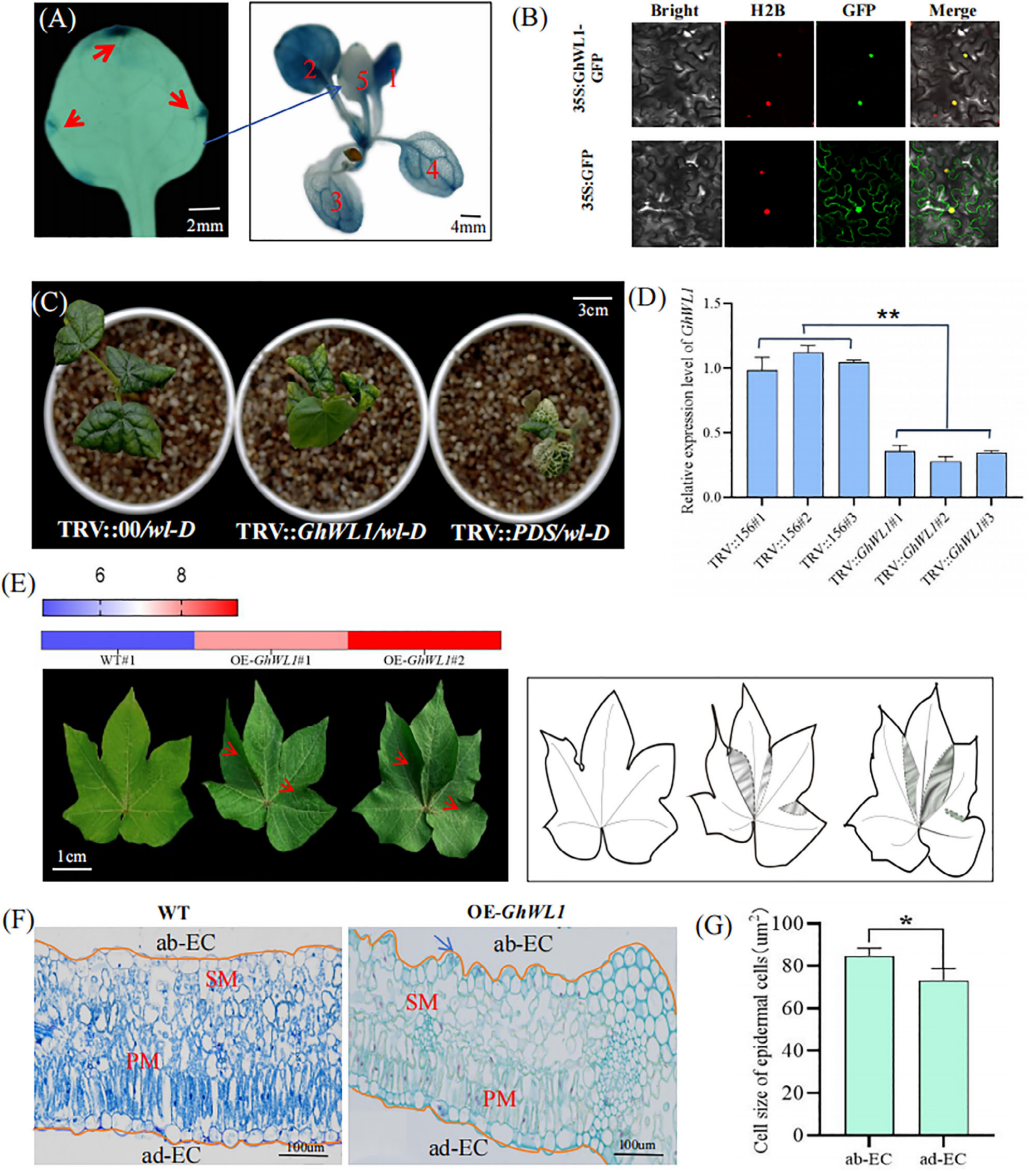
*GhWL1* expression dynamics and effects of *GhWL1*‐silencing on cotton leaf morphology. A) *GUS* reporter gene expression driven by the *GhWL1* promoter in transgenic *Arabidopsis thaliana* shows progressive expression patterns: early at leaf margin tips, intermediate in veins, and late in leaf tissues. Observations were conducted on ten plants. B) Subcellular localization analysis of *GhWL1* fused to GFP demonstrates its nuclear presence in *Nicotiana benthamiana* leaves, with free GFP serving as a control. H2B was used as a nuclear marker, and images were captured via confocal microscopy. C) Phenotypic comparison of *GhWL1*‐silenced plants (TRV:GhWL1) with positive (TRV:PDS) and negative controls (TRV:00) reveals morphological changes. D) RT‐qPCR analysis of GhWL1 expression in blank control and VIGS plants uses GhHistone3 as the internal normalization reference. Data are shown as mean ± SD for three independent experiments. **P < 0.01 (Student's *t*‐test). E) Overexpression of *GhWL1* (OE‐*GhWL1*) alters leaf phenotype, as shown in the schematic indicating positional folding. F) Toluidine blue‐stained paraffin sections of WT and OE‐*GhWL1* plants highlight cellular differences in ad‐EC, ab‐EC, PM, and SM layers. G) Epidermal cell size analysis in OE‐*GhWL1* leaves from (F) demonstrates a statistically significant increase. Data are presented as mean ± SD from ten independent experiments (n > 20). *P < 0.05 (Student's *t*‐test).

The gene *Ghicr24_D13G196500* was predicted to encode a protein within the NPR1 interact superfamily domain. Alignment results from NCBI revealed its similarity to the annotated XP_039028138 protein in *Hibiscus syriacus* (Figure S7, Supporting Information). Due to its distinctive impact on leaf morphology, it was designated WRINKLED‐LEAF 1 (*GhWL1*). Yeast activation assays confirmed that *GhWL1* functions as a transcription factor (Figure S8, Supporting Information). To characterize its expression pattern, a 1000‐bp segment upstream of the *GhWL1* start codon (ATG) was fused with the *β‐GLUCURONIDASE* (*GUS*) gene to create the *proWL1:GUS* construct. This construct was introduced into *Arabidopsis thaliana* ecotype Columbia‐0 (Col‐0) to generate stable transgenic lines. GUS staining revealed strong activity in the shoot apical region and leaf margins of juvenile leaves, expanding to vascular tissues and ultimately encompassing mature leaves (**Figure** **3**A; Figure S9, Supporting Information). These findings collectively suggest a dynamic expression of *GhWL1*, initiating predominantly at juvenile leaf margins and expanding throughout the leaf with maturation. Additionally, the subcellular localization of *GhWL1* was determined by fusing its 420‐bp coding sequence with green fluorescent protein (GFP) at the N‐terminus and transiently expressing this construct in *Nicotiana benthamiana* epidermal cells. Microscopic analysis confirmed nuclear localization of the *GhWL1*‐GFP fusion protein (Figure 3B)These observations indicate that GhWL1 is predominantly active in the nucleus, where it likely performs its regulatory functions related to leaf development.

To explore *GhWL1*’s role in cotton leaf development, virus‐induced gene silencing (VIGS) was employed to downregulate *GhWL1* in the *wl‐D* mutant. Silenced plants displayed normal leaf morphology, similar to wild type (WT) (Figure 3C,D), emphasizing the critical role of *GhWL1* in leaf morphogenesis. Additionally, transgenic lines overexpressing *GhWL1* (*OE‐GhWL1*) under the CaMV35S promoter were developed. These plants exhibited distinct phenotypes, including leaf curling at the margins and intensified green coloration, albeit with variations from the *wl‐D* phenotype (Figure 3E). RNA‐Seq and RT‐qPCR analyses revealed elevated *GhWL1* transcript levels in *OE‐GhWL1* lines, correlating with leaf abnormalities (Figure S10, Supporting Information). Paraffin sections of *OE‐GhWL1* plants showed irregular abaxial epidermal cell arrangement, identical to that in *wl‐D* mutants, further corroborating *GhWL1*’s role in leaf morphology regulation (Figure 3F,G).

### 2.4 GhH1 Collaborates with GhWL1 in Regulating Leaf Development

**Figure 4:**
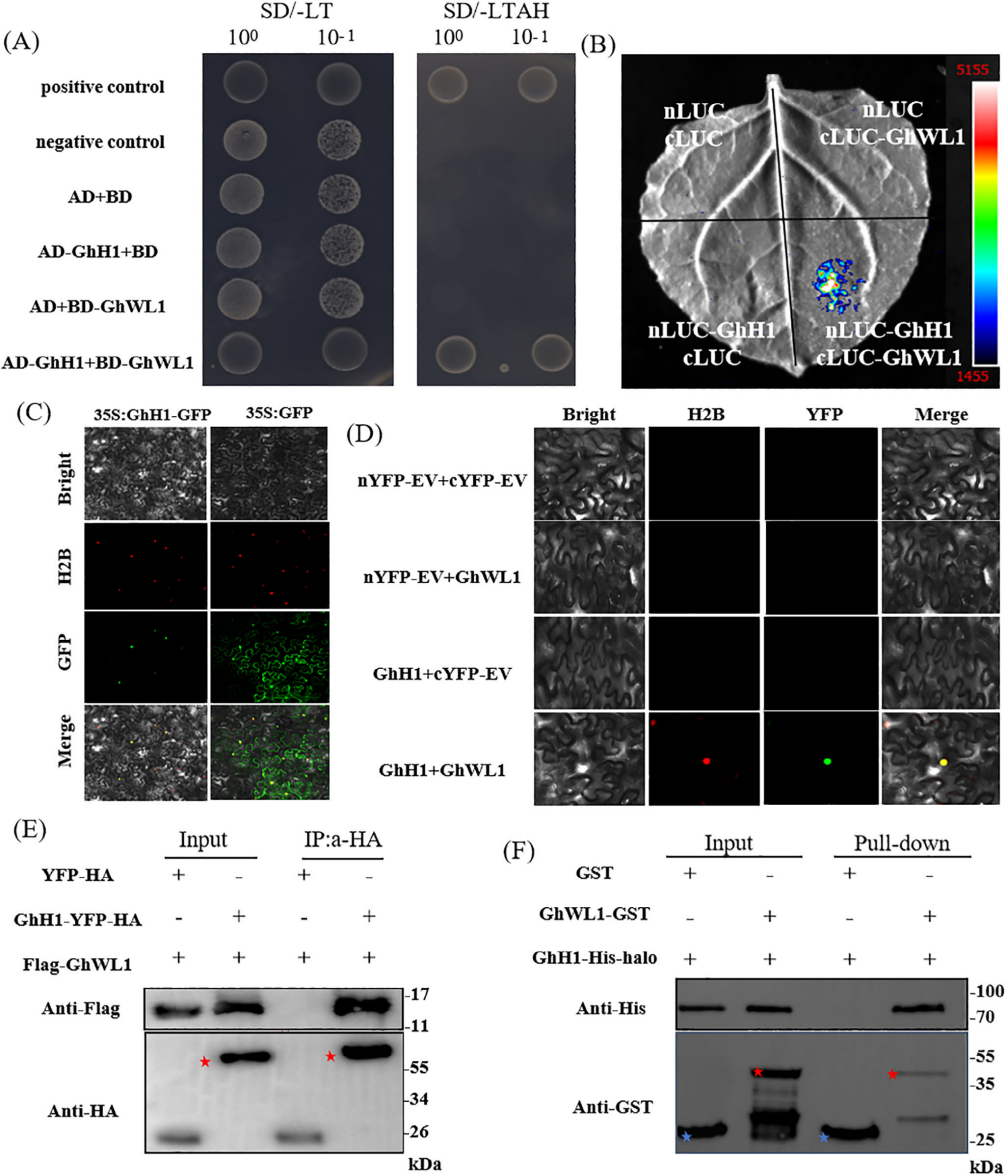
Interaction between GhWL1 and GhH1. A) Yeast two‐hybrid (Y2H) assay demonstrates the physical interaction between GhWL1 and GhH1. Yeast cells co‐expressing these proteins were diluted 10^0^ and 10^−1^ with water and inoculated on SD/‐Trp/‐Leu (SD/‐LT) and SD/‐Trp/‐Leu/‐His/‐Ade (SD/‐LTAH) selective media. Growth at 28 °C for two days confirmed their interaction. B) Luciferase Complementation Imaging (LCI) assay further validated the interaction between GhWL1 and GhH1. GhH1 was fused to the N‐terminal portion of LUC (nLUC), and GhWL1 was fused to the C‐terminal portion of LUC (cLUC). Agrobacteria carrying these constructs were co‐infiltrated into *Nicotiana benthamiana* leaves, with luminescence signals captured 48 h post‐infiltration. The color scale indicates LUC activity. This experiment was repeated three times with consistent results. C) Subcellular localization analysis revealed that GhH1, fused with GFP, localizes to the nucleus in *N. benthamiana* leaves. Free GFP served as the control, and H2B was used as a nuclear marker, visualized via confocal microscopy. D) Bimolecular fluorescence complementation (BiFC) assay confirmed the interaction between GhWL1 and GhH1 in *N. benthamiana*. E) Co‐immunoprecipitation (Co‐IP) analysis verified the interaction in *N. benthamiana*, with anti‐Flag and anti‐HA antibodies used for detection. The red asterisk denotes the target protein. F) Glutathione S‐transferase (GST) pull‐down assay further validated the interaction in vitro. GhWL1‐GST and GhH1‐His‐halo recombinant proteins were incubated with GST beads. Immunoblotting confirmed the pull‐down, with red asterisks identifying target proteins and blue asterisks indicating GST proteins.

To dissect the transcriptional network involving GhWL1, a yeast two‐hybrid (Y2H) screen using GhWL1 as bait identified 19 candidate proteins (Table S2, Supporting Information). Among these, GhH1 (Ghicr24_A06G177500), encoding a KNOX1 family transcription factor homologous to *Arabidopsis thaliana* STM (AT1G62360), directly interacted with GhWL1 (**Figure** **4**A; Figure S11, Supporting Information). Luciferase complementation imaging (LCI) confirmed the interaction between GhWL1 and GhH1 (Figure 4B). Subcellular localization experiments using GhH1‐GFP constructs showed nuclear localization, consistent with *GhWL1* (Figure 4C). Furthermore, bimolecular fluorescence complementation (BiFC) and co‐immunoprecipitation assays validated their interaction in plant cells (Figure 4D,E). GST pull‐down assays confirmed the direct interaction of GhWL1‐GST with GhH1‐His‐halo in vitro (Figure 4F).

**Figure 5:**
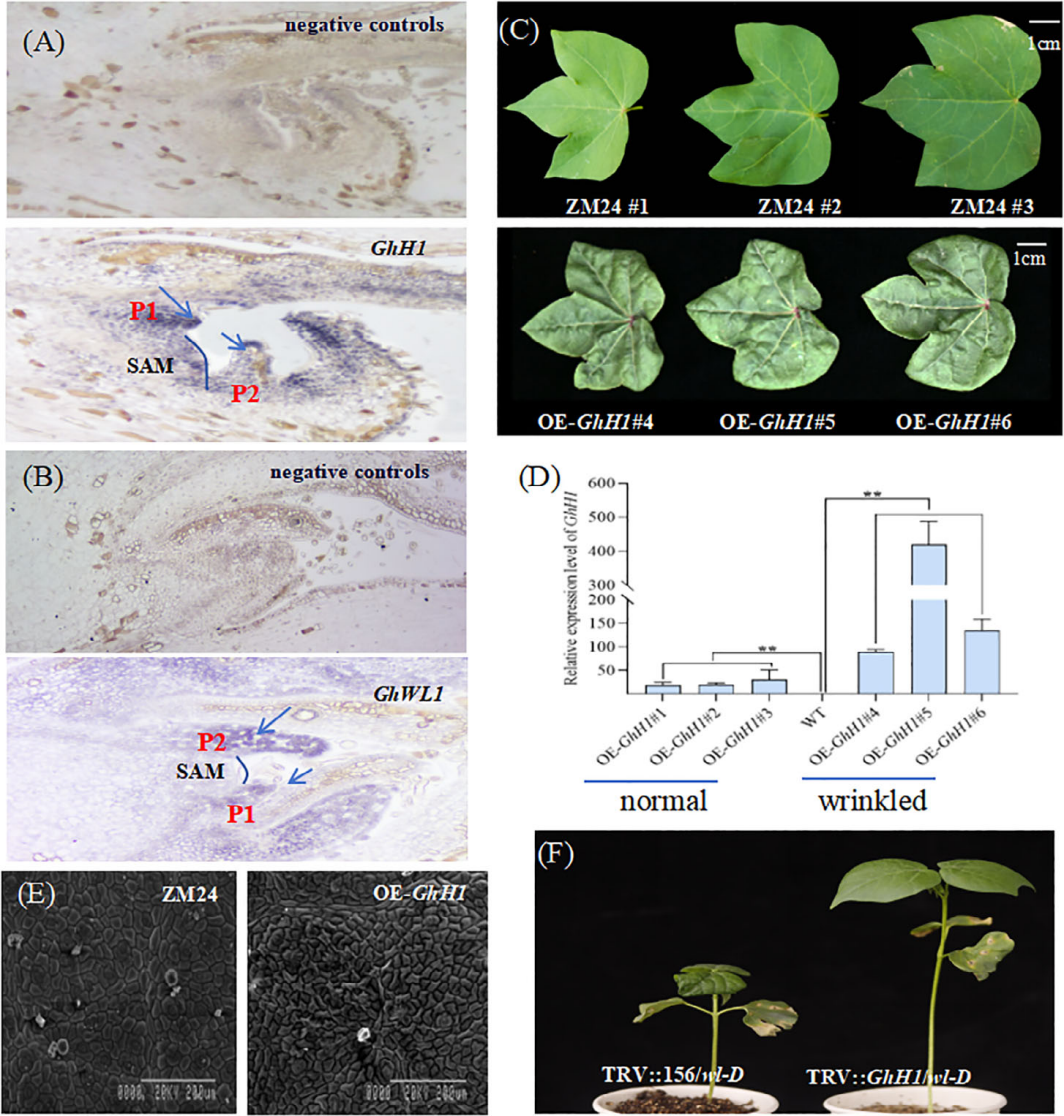
Functional analysis of *GhH1* in cotton leaf morphogenesis. A,B) RNA in situ hybridization localized *GhH1* and *GhWL1* expression. The blue line outlines the shoot apical meristem (SAM) contour between leaf primordia P1 and P2, while the blue arrow indicates the P1 and P2 leaf primordia. C) Overexpression of *GhH1* (OE‐*GhH1*) resulted in altered leaf morphology, with visible phenotypic differences. D) RT‐qPCR analysis quantified *GhH1* expression in OE‐*GhH1* lines. Data are presented as mean ± SD from three independent experiments. **P < 0.01 (Student's *t*‐test). E) SEM analysis revealed detailed changes in epidermal cell structure in OE‐*GhH1* leaves. F) Comparative phenotypic analysis showed clear distinctions between blank controls (*wl‐D*) and *GhH1*‐silenced plants.

Expression analysis of GhH1 using in situ hybridization demonstrated high expression levels in the shoot apical meristem (SAM) and leaf primordia, aligning with GhWL1 expression patterns (**Figure** **5**A,B). Overexpression of *GhH1* (OE‐GhH1) in transgenic lines produced wrinkled‐leaf phenotypes and sterility in plants with high GhH1 levels, whereas moderate overexpression resulted in normal leaves (Figure 5C,D). SEM analysis revealed cellular protrusions in *OE‐GhH1* leaves, akin to those observed in wl‐D (Figure 5E). To further investigate the genetic interplay between *GhWL1* and *GhH1*, VIGS experiments were conducted to suppress *GhH1* in *wl‐D* mutants. Silencing *GhH1* restored normal leaf morphology, indicating that *GhH1* collaborates with *GhWL1* in leaf morphogenesis (Figure 5F). These results suggest that the wrinkled‐leaf phenotype is contingent on the cumulative expression levels of *GhWL1* and *GhH1*, highlighting their synergistic role in regulating cotton leaf development.

### 2.5 GhWL1 and GhH1 Synergistically Regulate GA Signaling

**Figure 6:**
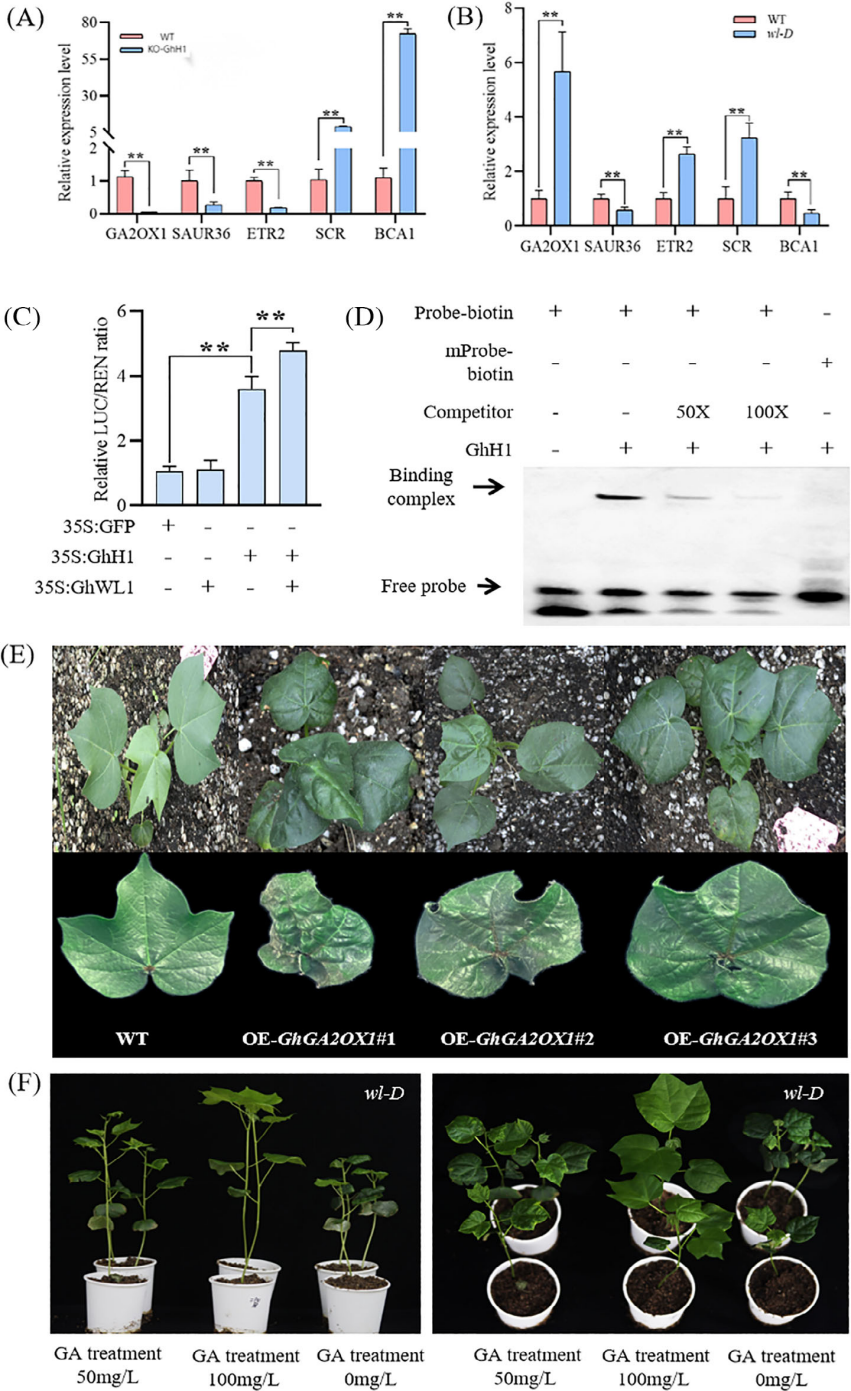
Transcriptional regulation by GhH1 via *GhGA2OX1* promoter binding. A,B) RT‐qPCR analysis assessed relative expression levels of putative downstream genes regulated by *GhH1* in WT, *wl‐D*, and KO‐*GhH1* plants. Histone (His) served as the reference gene. Data are shown as mean ± SD from three independent experiments. **P < 0.01 (Student's *t*‐test). C) A transient dual‐luciferase expression assay evaluated *GhH1* and *GhWL1* activity on the GhGA2OX1 promoter. Luminescence signals reflecting LUC activity were quantified, with data presented as mean ± SD (n = 5). **P < 0.01 (Student's *t*‐test). D) Electrophoretic mobility shift assay (EMSA) demonstrated GhH1's binding affinity to the *GhGA2OX1* promoter. A biotin‐labeled DNA probe confirmed the protein‐DNA interaction. The “+” symbol indicates interaction, while “‐” indicates the absence. E) Overexpression of *GhGA2OX1* (OE‐*GhGA2OX1*) altered leaf morphology compared to control plants. F) Application of varying concentrations of gibberellic acid (GA) to *wl‐D* leaves revealed dose‐dependent phenotypic changes.

To uncover the direct targets of GhH1, a DNA affinity purification sequencing (DAP‐seq) assay was conducted, identifying 137049 enriched peaks corresponding to 2388 genes (Table S3, Supporting Information). Among these, 11.34% of the peaks were located in promoter regions (up to 2 kb upstream of transcription start sites). Integration of RNA‐seq and DAP‐seq data revealed 66 genes that were both differentially expressed in wl‐D versus WT and KO‐GhH1 versus WT, and directly bound by GhH1 (Figure S13, Supporting Information). Five of these genes were implicated in four distinct hormone signaling pathways: auxin, gibberellic acid (GA), brassinosteroids (BRs), and ethylene. Among them, GhGA2OX1 (Ghicr24_A01G037600) exhibited the most pronounced differential expression in wl‐D versus WT and KO‐GhH1 versus WT comparisons, making it the primary focus of further analysis (**Figure** **6**A,B).

GA quantification in juvenile and adult leaves revealed a distinct pattern. In adult leaves, GA1 was undetectable, and GA4 levels remained unchanged in *wl‐D* and OE‐*GhH1* plants, but GA8 and GA34 concentrations were significantly higher than those in juvenile leaves. In juvenile leaves, however, GA1 and GA4 levels were significantly reduced (Figure S14, Supporting Information). To confirm the regulatory role of GhH1, electrophoretic mobility shift assays (EMSAs) and yeast one‐hybrid (Y1H) assays demonstrated that GhH1 directly binds to the *GhGA2OX1* promoter sequence, affirming its transcriptional regulation of this gene (Figure 6D; Figure S15, Supporting Information).

Given the interaction between GhWL1 and GhH1 and their shared role in regulating GhGA2OX1, it was hypothesized that GhWL1 and GhH1 collectively modulate GhGA2OX1, influencing leaf development. This hypothesis was tested using transient expression assays in N. benthamiana leaves, where co‐expression of p35S:GhWL1, p35S:GhH1, and pGA2OX1:LUC significantly increased LUC reporter activity driven by the GhGA2OX1 promoter (Figure 6C). Further co‐infiltration of GhWL1‐HA and GhH1‐FLAG in N. benthamiana leaves showed that GhWL1 did not affect GhH1 protein accumulation, suggesting that GhWL1 primarily enhances the transcriptional activity of GhH1 rather than its stability (Figure S16, Supporting Information). Genetic analyses further substantiated this regulatory interaction. Transgenic lines overexpressing GhGA2OX1 (OE‐GhGA2OX1) displayed a wrinkled‐leaf phenotype analogous to OE‐GhH1 plants (Figure 6E; Figure S17, Supporting Information). Application of exogenous GA restored normal leaf morphology in wl‐D, with the effect varying depending on the GA concentration (Figure 6F) (Figure 6F). These findings collectively demonstrate that GhWL1 and GhH1 synergistically regulate *GhGA2OX1* expression, which in turn modulates GA levels and leaf morphology, underscoring their pivotal role in coordinating GA signaling pathways for leaf development.

## 3 Discussion

**Figure 7:**
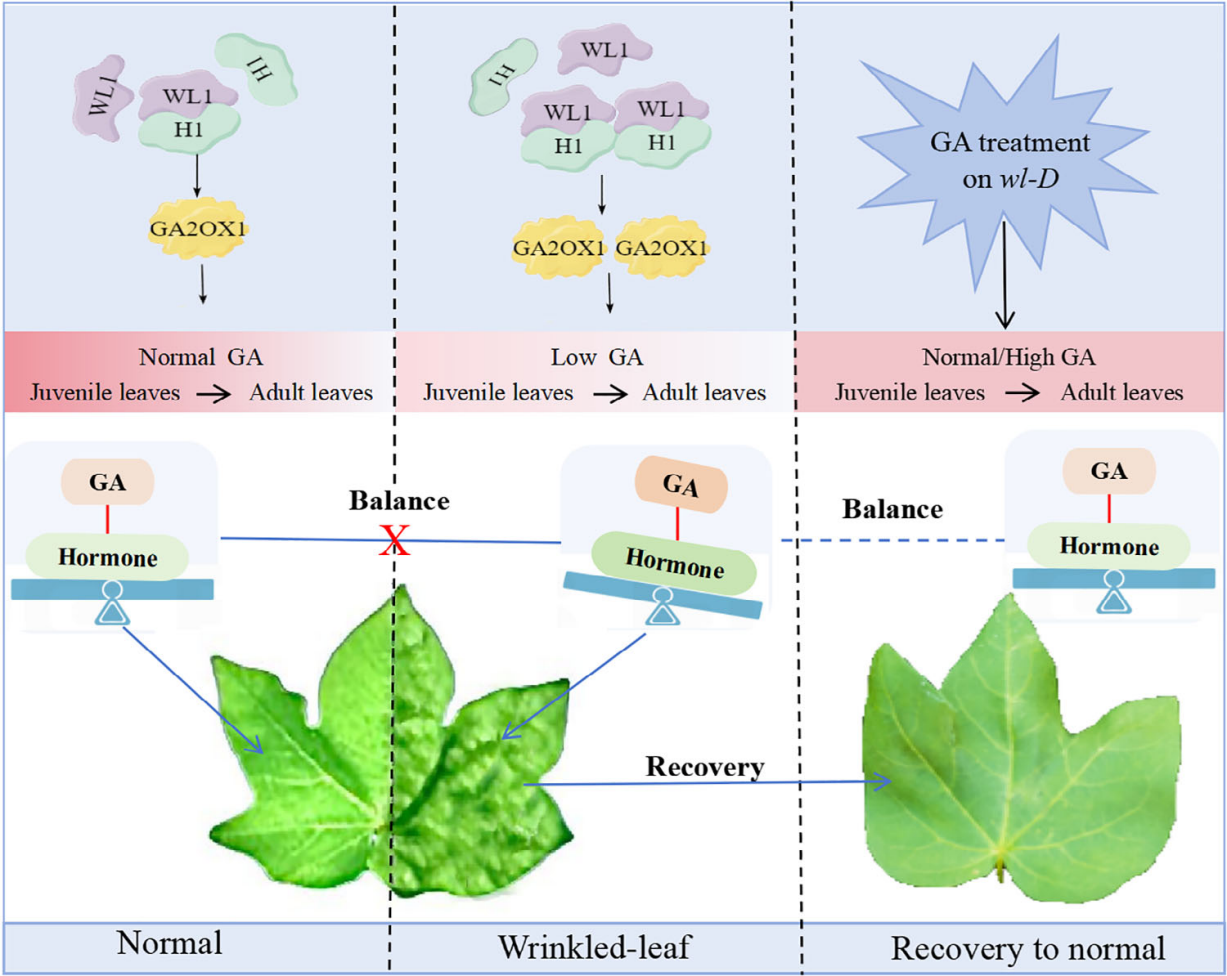
A working model for leaf development regulation. The color spectrum from pink to white indicates high to low GA levels. The leaves became normal after GA treatment in *wl‐D*.

The identification of key genes underlying wrinkled‐leaf morphology is a pivotal area of plant science, given its direct link to plant growth, development, and stress responses to environmental challenges such as drought and salinity. Unraveling the genetic basis of this phenotype is crucial for understanding plant stress adaptation mechanisms and enhancing resilience.^[^ ^32^, ^33^ ^]^ In this study, we identified a novel leaf shape mutant, wl‐D, through T‐DNA mutagenesis, characterized by wrinkled leaves (Figure 1A‐C). Chromosome walking enabled the isolation and cloning of GhWL1, a dominant gene regulating leaf development. Functional analyses, including gene knockout and overexpression experiments, confirmed GhWL1's role in the wrinkled‐leaf phenotype. Interestingly, while the wrinkled‐leaf phenotype in wl‐D was caused by GhWL1, the “yellow spot” feature was attributed to PPD1, a gene associated with the T‐DNA insertion, as verified by VIGS experiments (Figure S18, Supporting Information). Our findings further revealed that *GhWL1* interacts with *GhH1*, modulating its transcriptional activity rather than promoting over‐accumulation, thereby regulating *GhGA2OX1* expression. This, in turn, affects GA levels and leaf morphology (**Figure** **7**; Figure S16, Supporting Information). This regulatory module exemplifies how internal signals integrate to control leaf development and morphology.

Comparative analyses demonstrated significantly lower *GhWL1* expression levels in *OE‐GhWL1* plants compared to *wl‐D*. Notably, no overexpression lines exceeded the *wl‐D* mutant in expression levels. Given the low survival rate of *wl‐D* mutants, it is plausible that excessively high *GhWL1* expression levels may be lethal. This suggests that the milder phenotype observed in *OE‐GhWL1* plants may be due to the combined effects of *GhWL1* dosage and T‐DNA impacts on adjacent genes. While the *wl‐D* mutant displayed higher photosynthetic efficiency, its excessive *GhWL1* expression compromised agronomic traits such as fiber quality and yield. In contrast, moderate *GhWL1* expression in *OE‐GhWL1* plants improved photosynthetic efficiency, yield, and quality without inducing severe leaf deformities (Figure S19 and Table S4, Supporting Information). These insights emphasize the importance of fine‐tuning *GhWL1* expression to optimize leaf morphology and enhance plant performance under environmental stressors, offering opportunities for targeted breeding and sustainable agriculture.

Recent studies underscore the central role of *KNOX* family genes in leaf development, influencing cell division, expansion, and wall synthesis.^[^ ^34^, ^35^ ^]^ Specifically, STM genes are vital for maintaining apical meristem integrity.^[^ ^16^ ^]^ Variations in *STM* expression or copy number affect leaf morphology, with deviations from optimal levels leading to abnormal leaf development.^[^ ^36^ ^]^ In this study, GhH1 shared 65.97% sequence similarity with AtSTM, and phylogenetic analysis placed it within the AtSTM cluster, suggesting functional parallels (Figure S11, Supporting Information). VIGS‐mediated silencing of GhH1 in wl‐D mutants restored WT‐like leaf morphology (Figure 5F).

The *OE‐GhH1* lines exhibited two leaf phenotypes: wrinkled and normal. Quantitative expression analyses revealed significantly higher *GhH1* levels in wrinkled leaves compared to normal ones, indicating a dosage‐dependent effect on leaf development. This observation is reminiscent of dosage effects seen in *AGL24* modulating flowering time in *Arabidopsis* ^[^ ^37^ ^]^ and the distinct developmental impacts of strong stm‐1 versus weak stm‐2 alleles in Arabidopsis due to differences in STM expression.^[^ ^38^, ^39^ ^]^

Cotton contains 39 *KNOX* genes, 14 of which belong to the *KNOX1* subfamily (https://cottonfgd.net/). The minimal phenotypic changes in *GhH1* CRISPR lines suggest functional redundancy or complementary mechanisms within the genetic network governing leaf morphology (Figure S20, Supporting Information). This is akin to the lack of significant phenotypic changes in single *NF‐YC* mutants of *Arabidopsis*, where delayed flowering occurs only in double mutants.^[^ ^40^ ^]^ However, silencing *GhH1* in *wl‐D* successfully restored normal leaf morphology, underscoring its critical role in the aberrant phenotype. Future research is anticipated to further elucidate the precise roles and regulatory pathways of these genes in leaf development, paving the way for advancements in molecular breeding and agricultural productivity. This work highlights the potential for leveraging *GhWL1* and *GhH1* to develop cotton varieties with optimized leaf morphology, improved stress tolerance, and enhanced yield.

The remarkable plasticity of plant leaf development depends on intricate crosstalk between interconnected hormonal signaling pathways, which coordinate cellular activities and developmental processes.^[^ ^27^ ^]^ In this study, we demonstrated that the GhWL1‐GhH1 regulatory axis functions as a critical transcriptional hub, integrating signals from the IAA, BR, and GA pathways to regulate leaf development. Previous research has emphasized the importance of the auxin pathway in GhSTM‐mediated leaf development, highlighting the need for precise auxin distribution within specific spatiotemporal contexts.^[^ ^41^ ^]^ Auxin balances adaxial and abaxial cell growth, and its disruption can lead to curly leaf phenotypes.^[^ ^42^, ^43^, ^44^ ^]^

GA, known for promoting differentiation, regulate organ structures and developmental processes. During leaf development, GAs influence cell proliferation, expansion, and the complexity of leaf morphology.^[^ ^45^, ^46^ ^]^ Similarly, BRs stimulate cell elongation and differentiation, with mutations in BR biosynthesis or signaling pathways often resulting in aberrant leaf patterning.^[^ ^47^ ^]^ Our findings revealed that a gain‐of‐function mutation in *GhWL1* and *OE‐GhH1* plants altered the expression of multiple hormones signaling genes (Figures S21 and S22, Supporting Information). DAP‐seq analysis showed that *GhH1* binds directly to the promoters of key genes, including *SAUR36* (an auxin‐responsive gene), *GA2OX1* (Gibberellin 2‐beta‐dioxygenase 1), *SCR* (*SCARECROW*, involved in GA signal transduction), *ETR2* (Ethylene receptor 2), and *BCA1* (Beta carbonic anhydrase 1, a BR‐regulated gene). RNA‐seq data corroborated *GhH1*’s regulatory role in these pathways. Together, these findings underscore *GhH1*’s role as a pivotal transcriptional hub, orchestrating hormonal signaling and maintaining hormone homeostasis during leaf development. Exogenous application of GA to *wl‐D* plants with crumpled leaves restored normal leaf morphology, with higher GA concentrations producing more pronounced effects (Figure 6F). However, the absence of specific marker genes for GA content complicates mechanistic insights. Whether variations in leaf development stem from GA distribution or GA‐responsive genes warrants further investigation.

Leaf variation is a critical driver of species diversity, ecosystem resilience, and plant adaptation, while offering valuable insights into evolution and domestication processes. Originating as lateral branches, leaves have evolved diverse shapes and structures to adapt to varying habitats.^[^ ^3^ ^]^ For instance, thicker, smaller leaves in arid regions minimize water loss, while larger, thinner leaves in humid environments enhance photosynthetic efficiency and gas exchange.^[^ ^48^, ^49^ ^]^ Leaf morphology also plays a vital role in crop yield: compact leaves are ideal for dense planting, maximizing agricultural productivity.^[^ ^50^, ^51^, ^52^ ^]^

This study deepens our understanding of the genetic mechanisms underlying leaf shape diversity, particularly the interplay between *GhWL1*, *GhH1*, and GA pathways. By elucidating these pathways, we lay a theoretical foundation for future crop improvement strategies. Understanding the genetic factors and complex regulatory networks involved in leaf development advances our knowledge of plant evolutionary biology. Furthermore, these insights can inform targeted breeding and agricultural practices, enabling the cultivation of crop varieties with optimized traits for improved productivity and resilience.

## 4 Experimental Section

### Plant Materials and Growth Conditions

A wrinkled‐leaf mutant, designated as *wl‐D*, was identified from a T‐DNA insertion mutant collection derived from upland cotton (*Gossypium hirsutum*). For genetic analysis, the *wl‐D* mutant was crossed with the cultivar ZM24 to generate F_1_ and F_2_ progenies. Cotton plants, including mutants and crosses, were grown under natural field conditions in Anyang, Henan Province, China. Additionally, *Arabidopsis thaliana* (ecotype Col‐0) and *Nicotiana benthamiana* were cultivated in controlled environments maintained at 22 °C, 60% relative humidity, and long‐day conditions (16 h light/8 h dark) under white light illumination.

### Isolation of T‐DNA Flanking Sequences Through Thermal Asymmetric Interlaced PCR

T‐DNA flanking sequences were amplified using the TAIL‐PCR method following the protocol of Liu et al.^[^ ^53^ ^]^ Genomic DNA from the mutant served as the template. Three nested primers (RSP1, RSP2, RSP3; LSP1, LSP2, LSP3) and nine arbitrary degenerate primers (SP1, SP2, SP3, etc.) were used (Table S1, Supporting Information). PCR products were purified using the EZNA Gel Extraction Kit (Omega Bio‐Teck, Norcross, GA, USA) and sequenced. The T‐DNA integration sites were identified by aligning the genomic sequences between the WT and the T‐DNA locus using the BLAST tool on the CottonFGD platform.

### Genetic Analysis and Population Construction

The inheritance pattern of the wrinkled‐leaf trait was analyzed by crossing the *wl‐D* mutant with ZM24 to produce F_1_ plants. Self‐pollination of F_1_ plants yielded the F_2_ population. Phenotypes (wrinkled vs normal leaves) were recorded for parental lines, F_1_ plants, and F_2_ progenies. Segregation ratios were calculated to elucidate the genetic basis of the trait. Genomic DNA was extracted from juvenile leaves of F_2_ individuals using the CTAB method.

### RNA Sequencing and Data Analysis

Leaf samples were collected from four plants, with three biological replicates per sample. Total RNA was extracted and sequenced on the Illumina 2500 platform (Illumina, San Diego, CA, USA) by the Beijing Genomics Institute (BGI, Shenzhen, China). RNA‐seq data were aligned to the TM‐1 (AD1) cotton genome (NAU‐NBI assembly v1.1).^[^ ^54^ ^]^ Gene expression levels were normalized to FPKM values using Stringtie. Differentially expressed genes (DEGs) were identified using DESeq2 (https://workshop.veupathdb.org/bop/pdfs/beginner_DeSeq2.pdf) with criteria of fold change >2.0 and adjusted P < 0.01. Gene ontology (GO) enrichment analysis was performed with (https://david.ncifcrf.gov), while Kyoto Encyclopedia of Genes and Genomes (KEGG) enrichment analysis was performed on the Novogene platform (https://magic.novogene.com).

### Quantitative Reverse Transcriptase PCR

Total RNA was extracted using the Plant RNAprep Pure Kit (Tiangen, Beijing, China) and treated with DNase I. cDNA synthesis was carried out using the PrimeScript RT reagent kit (Takara, Tokyo, Japan). RT‐qPCR was performed with the SuperReal PreMix Plus Kit (SYBR Green; Tiangen). Relative expression levels were calculated using the 2−^ΔΔCT^ method.^[^ ^55^ ^]^ Primers are listed in Table S1 (Supporting Information), with *Actin2* and *UBQ* serving as endogenous controls.

### Phylogenetic Analysis of WL1 Proteins

Homologous WL1 proteins were retrieved from the Arabidopsis Information Resource (http://www.arabidopsis.org) and NCBI (https://www.ncbi.nlm.nih.gov/) databases. Full‐length protein sequences were aligned using ClustalW, and a phylogenetic tree was constructed via the maximum‐likelihood method with 1000 bootstrap replicates.

### Histochemical *β*‐GLUCURONIDASE (GUS) Analysis

A 2.0 kb promoter region of *GhWL1* was amplified and cloned into p35S::GUS, replacing the 35S promoter. GUS expression in cotton was assessed histochemically using X‐Gluc as a substrate.^[^ ^56^ ^]^ Leaf and stem segments of *Arabidopsis* were incubated in X‐Gluc solution (0.2 M Na_3_PO_4_, 1 mg mL^−1^ X‐Gluc, 0.1% Triton X‐100, 10 mM EDTA, pH 7.0) at 37 °C for 4 h. Samples were decolorized with 75% ethanol for visualization.

### Subcellular Localization of *GhWL1*

The full‐length coding sequence (CDS) of *GhWL1*, excluding the stop codon, was amplified from ZM24 with specific primers (Table S1, Supporting Information). This sequence was cloned into the pCAMBIA2300‐GFP vector at the *PacI* and *XbaI* sites using homology recombination, generating a 35S:GhWL1‐GFP fusion protein for transient expression. The recombinant plasmid, along with the control plasmid (35S:GFP), was introduced into *Agrobacterium tumefaciens* strain GV3101. Leaves of 4‐week‐old *Nicotiana benthamiana* plants were infiltrated with *A. tumefaciens* carrying the GhWL1 construct. After 48 h, epidermal cells from infiltrated leaves were observed under an Olympus FV1200 confocal microscope (Olympus, Tokyo, Japan) to determine the subcellular localization of the GhWL1‐GFP fusion protein. Detailed information on the primers used is available in Table S1 (Supporting Information).

### Scanning Electron Microscopy

SEM was employed to examine the leaf surfaces of mutant and WT cotton plants, following a previously described protocol with slight modifications to suit the specific characteristics of the samples.

### RNA In Situ Hybridization

In situ hybridization was carried out as per Coen et al.,^[^ ^57^ ^]^ with minor adjustments. Digoxigenin‐labeled sense and antisense probes targeting the full‐length cDNAs of *GhWL1* and *GhH1* were used. Eight‐micrometer sections from shoot apices of 4‐week‐old seedlings were hybridized, and signals were visualized with an Olympus BX63 microscope equipped with differential interference contrast (DIC) imaging.

### Virus‐Induced Gene Silencing (VIGS)

Virus‐induced gene silencing (VIGS) was employed to downregulate *GhWL1* expression in cotton.^[^ ^58^ ^]^ A 200–300 bp fragment of the *GhWL1* CDS was amplified and cloned into the pTRV vector at the *XbaI* and *BamHI* sites via homology recombination. This construct was transformed into *Escherichia coli* DH5α cells for validation before introducing it into *A. tumefaciens* strain GV3101. *A. tumefaciens* cultures carrying various constructs (pTRV:PDS as a positive control, pTRV:00 as a negative control, pYL192 as an auxiliary vector, and pTRV:GhWL1 for VIGS) were infiltrated into the cotyledons of 7‐day‐old *wl‐D* mutant seedlings. After 24 h of dark incubation, plants were transferred to a greenhouse. Leaf samples were collected two weeks later for RT‐qPCR analysis and phenotypic assessment to confirm gene silencing efficacy.

### Cotton Transformation

The complete CDSs of *GhWL1*, *GhH1*, and *GhGA2OX1* were amplified from ZM24‐derived cDNA and cloned into the WMV067 vector pre‐digested with *SalI* and *BamHI*, generating 35S:GhWL1, 35S:GhH1, and 35S:GhGA2OX1 expression vectors. These constructs were introduced into ZM24 plants via *Agrobacterium*‐mediated transformation, as described by Ge et al.^[^ ^59^ ^]^ Primers used for transgenic plant analysis are listed in Table S1 (Supporting Information).

### DNA Affinity Purification Sequencing

For DAP‐seq, genomic DNA was extracted from juvenile cotton leaves, fragmented, and ligated to truncated Illumina TruSeq adaptors. HALO‐tagged transcription factors (TFs) were synthesized in vitro, immobilized on Magne HALO‐Tag beads, and incubated with the fragmented genomic DNA library. After washing, DNA was eluted, amplified with indexed TruSeq primers, and sequenced on an Illumina HiSeq 2500 platform, yielding 100‐bp single reads. The ampDAP‐seq library was prepared via PCR with Phusion High‐Fidelity DNA Polymerase.^[^ ^60^ ^]^ Reads were mapped to the TM‐1 genome, and peaks were identified using the GEM peak caller.^[^ ^61^ ^]^

### Yeast Two‐Hybrid Assays

Y2H assays were performed as described by Xiao et al.^[^ ^62^ ^]^ The CDS of *GhWL1* was cloned into the pGBKT7 vector (*EcoRI* and *BamHI* sites), while the full‐length *GhH1* cDNA was cloned into the pGADT7 vector (*EcoRI* and *SacI* sites) via homology recombination. These constructs were co‐transformed into *Saccharomyces cerevisiae* Y2H Gold. Protein‐protein interactions were assessed on selective media with varying stringencies: low‐stringency (‐Leu/‐Trp) and high‐stringency (‐Ade/‐His/‐Leu/‐Trp). Primers used in this experiment are detailed in Table S1 (Supporting Information).

### Yeast One‐Hybrid Assays

Y1H assays were conducted according to the manufacturer's instructions (Clontech Laboratories, Mountain View, CA, USA). A 200‐bp promoter fragment of *GhGA2OX1* (pGhGA2OX1) and its mutant version (*mpGhGA2OX1*) were cloned into the pAbAi vector with an AbA reporter gene, generating pAbAi‐pGhGA2OX1 and pAbAi‐mpGhGA2OX1, respectively. The coding region of **GhH1** was cloned into the pGADT7 vector (*EcoRI* and *SacI* sites) to produce AD‐GhH1, which was expressed in Y1H Gold yeast strains harboring pAbAi‐pGhGA2OX1 or pAbAi‐mpGhGA2OX1. Protein‐DNA interactions were determined by monitoring yeast growth on a medium containing 200 µg L⁻¹ AbA.

### Luciferase Complementation Imaging Assay

The LCI assay was conducted to explore protein‐protein interactions between GhWL1 and GhH1. The coding sequences of *GhWL1* and *GhH1* were PCR‐amplified using primers listed in Table S1 (Supporting Information). *GhWL1* was fused in‐frame with the C‐terminus of luciferase (cLUC) in the 35S:cLUC vector at the *Kpn*I and *Sac*I sites, while *GhH1* was similarly fused in‐frame with the N‐terminus of luciferase (nLUC) in the 35S:nLUC vector using the same restriction sites via homology recombination. The resulting constructs were introduced into *Agrobacterium tumefaciens* strain GV3101 and infiltrated into *Nicotiana benthamiana* leaves. The plants were incubated under a 16‐h light/8‐h dark cycle for two days post‐infiltration. Luciferase signals were visualized by spraying the abaxial leaf surfaces with 1 mM luciferin, followed by a 10‐min dark incubation, and detection using a Tanon 5200 Multi chemiluminescent imaging system (Tanon, Shanghai, China). Additionally, firefly luciferase (LUC) and Renilla luciferase (REN) activities were quantified using a dual‐luciferase reporter assay system (Promega, Madison, WI, USA).

### Glutathione S‐Transferase Pull‐Down Assay

The GST pull‐down assay was performed to confirm interactions between GhWL1 and GhH1. GhH1 (excluding its signal peptide) was cloned into the pGEX‐4T‐3 vector to produce GST‐tagged bait protein, while GhWL1 (without its signal peptide) was cloned into the pET32a vector to express a His‐tagged prey protein. Both constructs were expressed in *Escherichia coli* strain BL21 (DE3). A GST‐expressing pGEX‐4T‐3 vector served as a negative control. Bait and prey proteins were incubated at 4 °C for 6 h before purification using glutathione‐conjugated agarose beads (GE Healthcare, San Ramon, CA, USA). Protein complexes were separated by 12% SDS‐PAGE and analyzed via immunoblotting with anti‐GST and anti‐His antibodies (Proteintech, Rosemont, IL, USA).

### Co‐Immunoprecipitation

For Co‐IP assays, *GhH1* and *GhWL1* coding sequences were cloned into the QBV3 vector at the *EcoR*V site to create QBV3‐GhH1 and QBV3‐GhWL1 constructs. These were recombined into pEarlyGate101‐HA and pEarlyGate100 vectors using the Gateway LR Clonase II Enzyme Mix (Thermo Fisher Scientific, Waltham, MA, USA), generating GhH1‐YFP‐HA and GhWL1‐Flag constructs. The constructs were introduced into *A. tumefaciens* GV3101 and infiltrated into *N. benthamiana* leaves. After three days, proteins were extracted with RIPA buffer and incubated with HA‐agarose‐conjugated beads (Sigma, St. Louis, MO, USA) at 4 °C for 3 h to isolate protein complexes. Immunoprecipitated proteins and input controls were analyzed by 12% SDS‐PAGE, followed by Western blotting with anti‐Flag (1:1000, Sigma–Aldrich) and anti‐HA (1:1000, Abcam, Cambridge, UK) antibodies.

### Biomolecular Fluorescence Complementation Assays

BiFC assays were employed to assess protein‐protein interactions in living cells. GhWL1 and GhH1 proteins were fused to complementary fragments of YFP via homology recombination and transiently expressed in *N. benthamiana* leaves using *A. tumefaciens* infiltration. After 48 h, fluorescence signals were detected using a confocal laser‐scanning microscope. Each experiment was conducted using six leaves, with three independent replicates to ensure reproducibility.

### Electrophoretic Mobility Shift Assay

EMSA was performed to study DNA‐protein interactions involving GhH1. The *GhH1* CDS was expressed with a His‐tag in *E. coli* BL21. Biotin‐labeled probes were prepared by annealing sense and antisense primers, with the 5′ ends of the sense primers labeled with biotin. EMSAs were conducted using the LightShift Chemiluminescent EMSA Kit (Thermo Fisher Scientific), and DNA‐protein binding was identified by shifts in mobility on SDS‐PAGE. Fluorescence‐labeled DNA bands were visualized using a Tanon‐4600 instrument (FujiFilm, Tokyo, Japan). Sequences of the probes used are detailed in Table S1 (Supporting Information).

### Protein Extraction and Western Blot Analysis

GhWL1 and GhH1 proteins, fused to complementary GFP or GFP‐HA fragments, were transiently expressed in *N. benthamiana* leaves via *A. tumefaciens*‐mediated infiltration. Protein extracts were prepared two days post‐infiltration for Western blot analysis. Proteins were separated on a 10% Bis‐Tris SDS‐PAGE gel (Sangon Biotech, Shanghai, China) at 90 V for 2 h and transferred to PVDF membranes (Merck Millipore, Tullagreen, Ireland) at 200 mA for 2 h. Membranes were blocked with 5% skim milk for 1.5 h at room temperature, followed by overnight incubation at 4 °C with primary antibodies: anti‐HA (1:5000; MBL, Tokyo, Japan) or anti‐Flag (1:5000; MBL). After washing, membranes were incubated with HRP‐conjugated secondary antibodies (1:5000; MBL) for 1.5 h at room temperature. Signals were developed using Immobilon Western Chemiluminescent HRP Substrate (Millipore, Burlington, MA, USA) and captured using the Chemidoc XRS+ system (Bio‐Rad, Hercules, CA, USA).

### Measurements of Endogenous Gibberellins

GAs were quantified by MetWare (http://www.metware.cn/) using the AB Sciex QTRAP 6500 LC‐MS/MS platform (SCIEX, Framingham, MA, USA), with minor modifications based on the method described by Urbanová et al.^[^ ^63^ ^]^ Briefly, 50 mg of cotton tissue was homogenized in 1 mL of ice‐cold 80% (v/v) acetonitrile containing 5% (v/v) formic acid. Internal GA standards, sourced from Olchemim Ltd. (Olomouc, Czech Republic) and Sigma, were added to the extraction buffer. The samples were extracted overnight at 4 °C using a Stuart SB3 benchtop rotator (Bibby Scientific, Staffordshire, UK). Following extraction, the homogenates were centrifuged, and the resulting supernatants were purified using ion‐exchange solid‐phase extraction (SPE) cartridges (Waters, Milford, MA, USA). The purified samples were analyzed using ultra‐high‐performance liquid chromatography‐tandem mass spectrometry (UHPLC‐MS/MS; Micromass, Cheshire, UK). Gibberellins were detected in multiple‐reaction monitoring mode, tracking the transition of the [M‐H] precursor ion to the corresponding product ion. Data acquisition and analysis were performed using Analyst 1.6.3 and Multiquant 3.0.3 software (AB Sciex).

### Effects of GA Treatment on *wl‐D*

To examine the effects of gibberellic acid (GA) on leaf development in the wl‐D mutant, two‐leaf‐stage seedlings were cultivated under controlled growth chamber conditions with a 14‐h/10‐h light/dark cycle at 30 °C. GA3 solution was applied to the leaves using a sponge ball, with applications performed at the two‐leaf stage and repeated after 10 days. Phenotypic changes in leaf development were observed and recorded at subsequent stages to assess the impact of the GA treatment.

### Measurement of SPAD Values in the Field

SPAD values were measured in 90‐day‐old cotton plants to assess leaf chlorophyll content. Leaves were sampled at three canopy levels: upper, middle, and lower. For each sampling, three upper leaves were randomly selected, and four measurement points per leaf were recorded using a SPAD chlorophyll meter (Konica Minolta, Tokyo, Japan). The SPAD readings for each leaf were averaged to represent the greenness at the corresponding canopy position.

### Statistical Analysis

Statistical analyses were conducted using Microsoft Excel, where mean values and standard deviations (SD) were calculated. The significance of differences between groups was determined using the Student's *t*‐test.

## Conflict of Interest

The authors declare no conflict of interest.

## Author Contributions

G.‐X.Y., L.‐F.G., and K.J., designed and supervised the research; W.Y.,W.P., L.‐M.H., W.X., C.‐Y.L, and Q.‐W.Q. performed the experiments; G.‐X.Y., Z.H, and Z.‐J.J. wrote the manuscript. All authors discussed the results and commented on the manuscript.

## Supporting information



Supporting Information

Supporting Information

## Data Availability

The data that support the findings of this study are available in the supplementary material of this article.
