# Supplementary material for: The GhWL1‐GhH1‐GhGA2OX1 Transcriptional Module Regulates Cotton Leaf Morphology
Source: Adv Sci (Weinh). 2025 Apr 30;12(20):2410783. doi: 10.1002/advs.202410783 (PMC12120715; doi:10.1002/advs.202410783)
Supplement: Supplementary file 1 — Supporting Information [file ADVS-12-2410783-s002.docx]

**Supporting Information**

**The GhWL1-GhH1-GhGA2OX1 transcriptional module regulates cotton leaf morphology**

Jingjing Zhan^1#^, Xiaoshuang Zhang^4#^, Ye Wang^1#^, Hang Zhao^1,3#^, Yu Chu^1^, Peng Wang^1^, Yanli Chen^1^, Xi Wei^1^, Wenqiang Qin^1^, Menghan Liu^1^, Jie Kong^2*^, Fuguang Li^1*^, Xiaoyang Ge^1*^

^
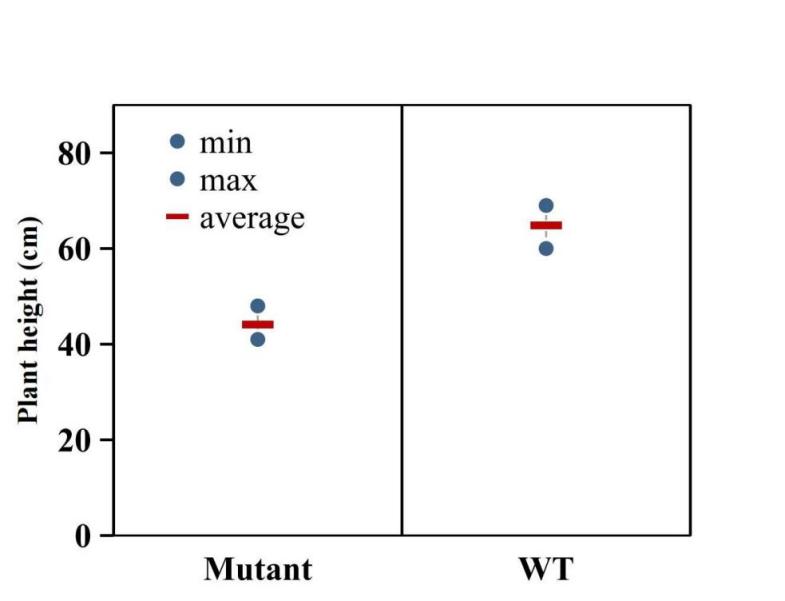
^

**Supplementary Figure 1. Plant height (PH) of cotton *wl-D* mutant and wild-type ZM24.** Data are represented as mean ± SD based on three independent experiments with n > 20.


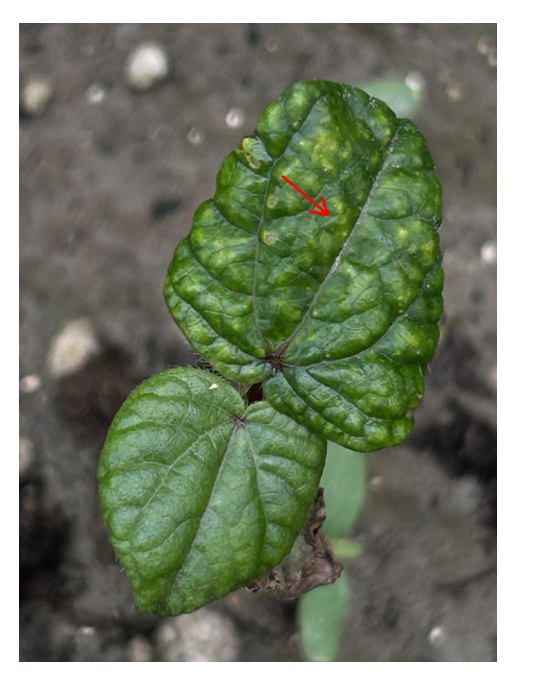


**Supplementary Figure 2. Appearance of yellow spots on the leaves of the cotton *wl-D* mutant.**


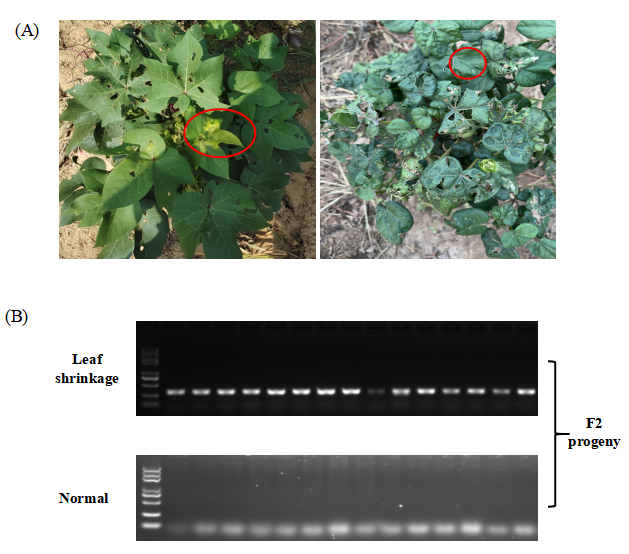


**Supplementary Figure 3.** **Co-segregation analysis of T-DNA insertions with specific phenotypes in the F_2_ generation.** (A) Phenotypic observation of kanamycin-sensitive cotton plants, where normal leaves exhibited sensitivity to kanamycin. (B) PCR analysis of the F2 population using kanamycin-specific primers.


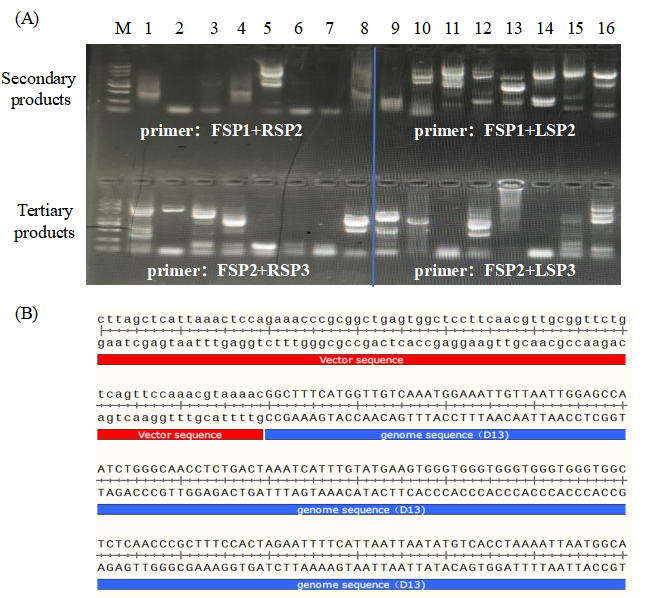


**Supplementary Figure 4.** **Characterization of flanking sequences in the *wl-D* mutant using leaf-derived DNA.** (A) Agarose gel electrophoresis results showing secondary products (first row) and tertiary products (second row) derived from specific samples. Lane details: M, molecular weight marker; 1–16, amplification products using templates from ZM24 with primers SP1+LSP1, SP2+LSP1, etc. (B) Schematic representation of T-DNA integration within the cotton genome, supplemented by sequence alignment data.


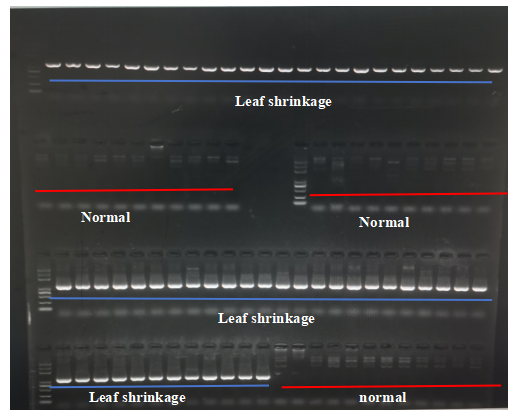


**Supplementary Figure 5. Genotypic analysis of the F_2_ population.** Primers P1a and P2 were used to enable genotyping of the F_2_ population. The presence of specific bands indicates T-DNA integration. M, Trans2K plus DNA Marker.


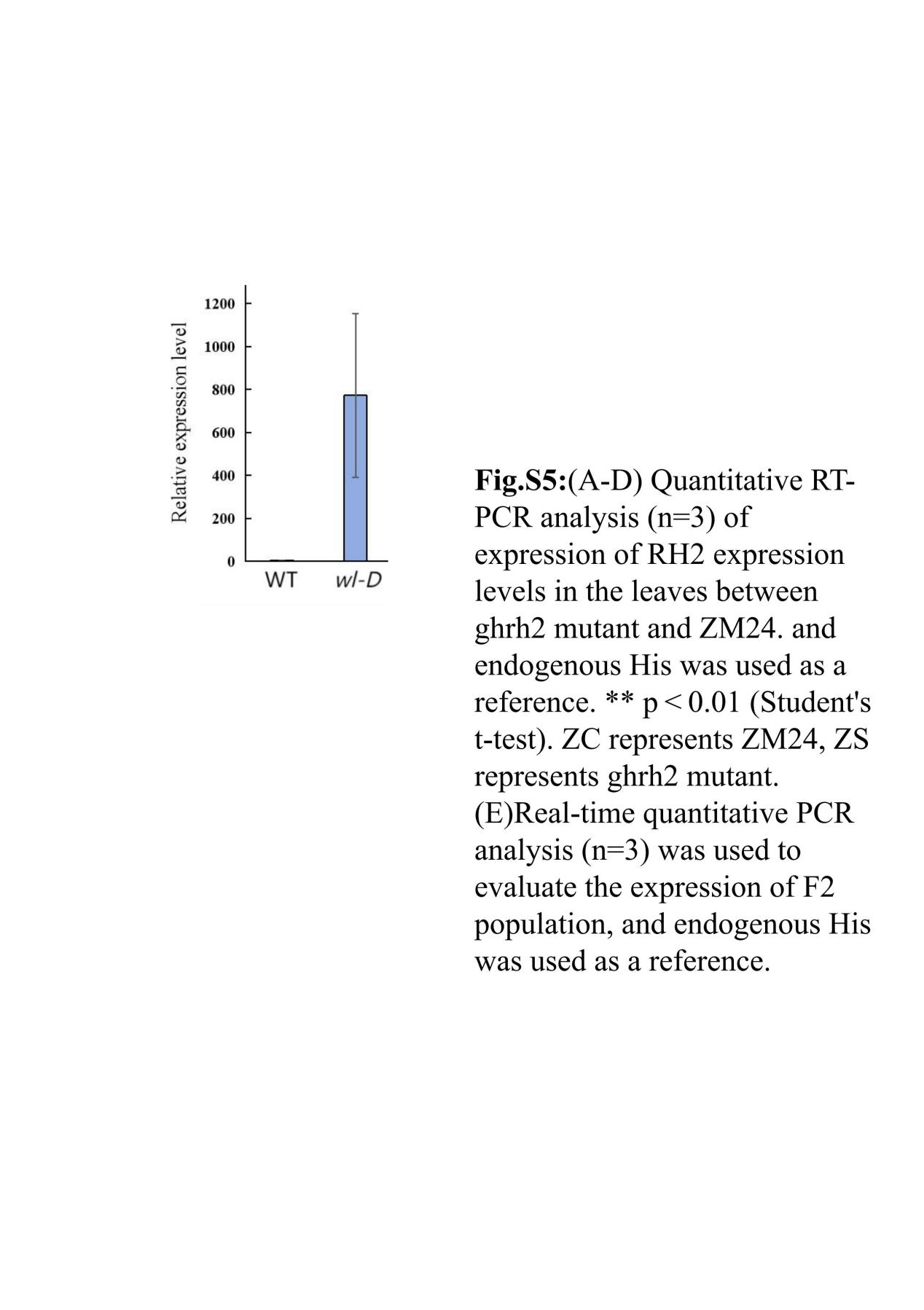


**Supplementary Figure 6.** **Quantitative reverse transcriptase PCR (RT-qPCR) analysis of *GhWL1* expression in the leaves of *wl-D* mutants and ZM24.** Histone (His) served as the endogenous control. Values are expressed as mean ± SD (n = 3). ** *P*< 0.01 (Student's *t-*test).


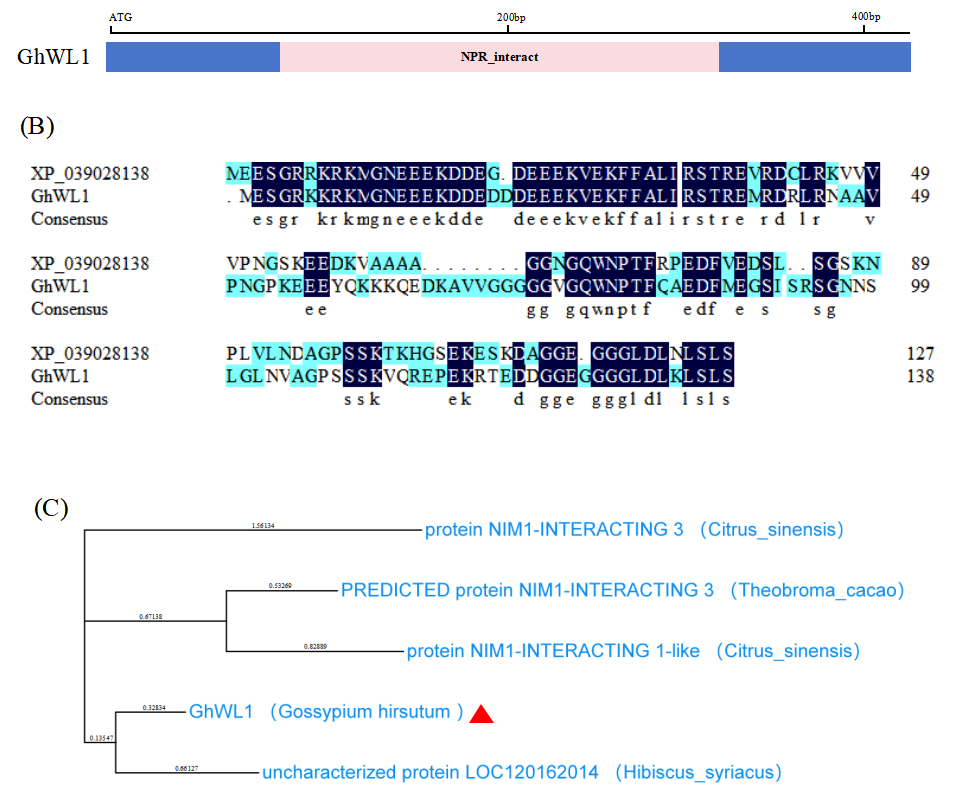


**Supplementary Figure 7. *GhWL1* gene information.** (A) Schematic diagram of the gene structure. (B) Sequence alignment of *GhWL1* with orthologs from *Hibiscus syriacus* (*XP_0390281138*), highlighting identical amino acids with a black background. (C) Phylogenetic analysis of *GhWL1* and its homologs, with bootstrap values shown near nodes. Triangles represent protein sequences from cotton. Branch lengths indicate relative amino acid variation rates, with bootstrap values derived from 1000 replicates.


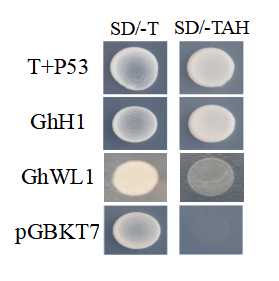
**Supplementary Figure 8. Transactivation activity assay of GhH1 and GhWL1.** SD/-T: SD/-Trp, SD/-TAH: SD/-Trp/-His/-Ade.


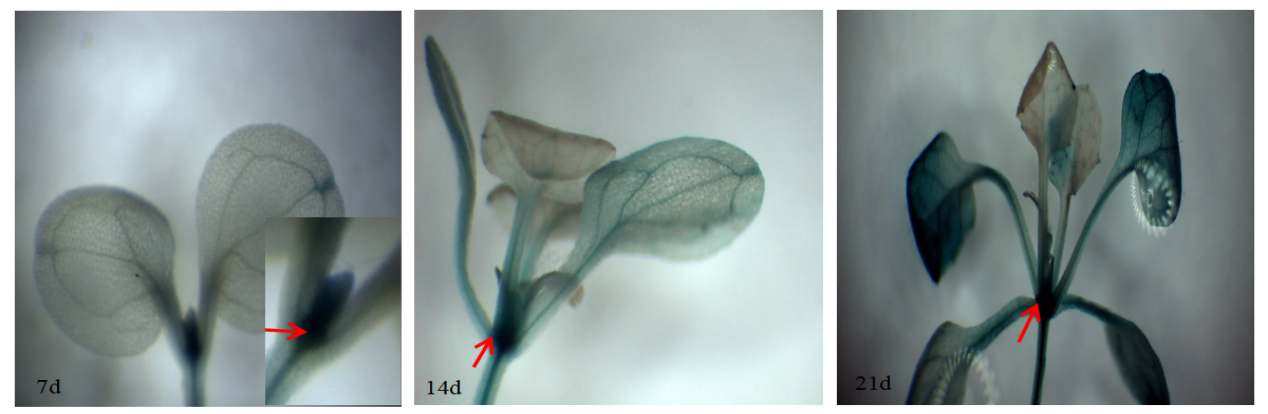


**Supplementary Figure 9. *GUS* reporter gene expression under the *GhWL1* promoter in transgenic *Arabidopsis* *thaliana* at various developmental stages.**


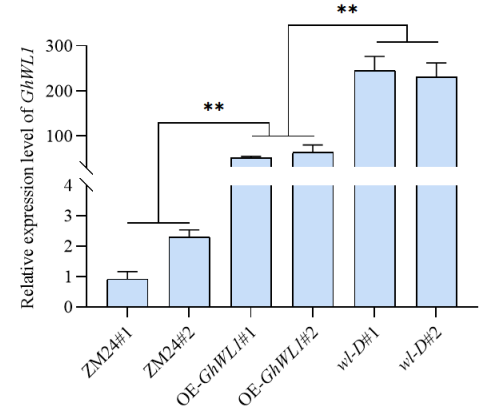


**Supplementary Figure 10.** Quantitative reverse transcriptase PCR (RT-qPCR) analysis evaluating the relative expression level of putative downstream genes regulated of GhWL1 in WT, *wl-D,* and OE-*GhGA2OX1*. Endogenous *Histone* (*His*) was used as a reference gene. Data are presented as mean ±SD for three independent experiments. ***P* < 0.01 (Student's *t*-test).


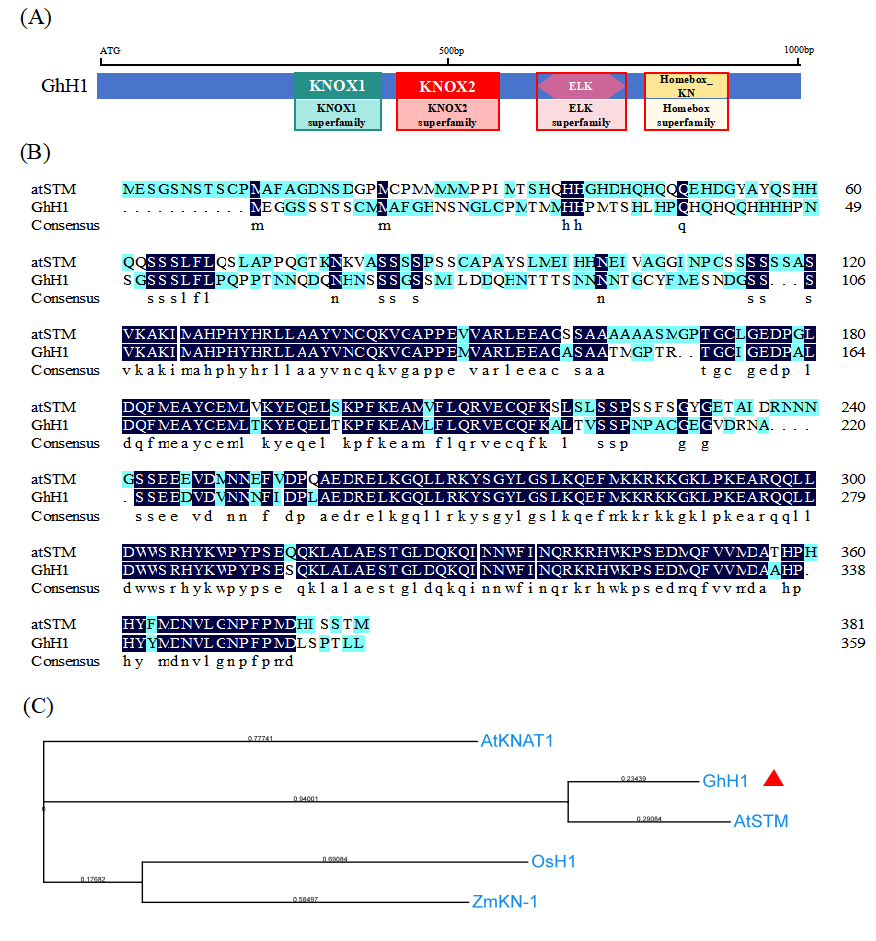


**Supplementary Figure 11. *GhH1* gene information.** (A) Schematic representation of gene structure. (B) Sequence alignment of *GhH1* with orthologs from *Arabidopsis thaliana* (*AtSTM*), with identical amino acids highlighted in black. (C) Phylogenetic analysis of *GhH1* and its homologs, including bootstrap values and relative branch lengths derived from 1000 replicates.


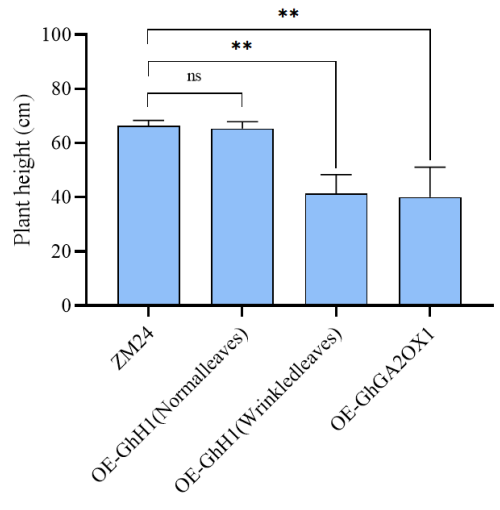


**Supplementary Figure 12. Plant height (PH) of cotton OE-*GhH1*, OE-*GhGA2OX1,* and wild type ZM24.** ns indicates no significant difference, ***P* < 0.01 (Student's *t-test*).


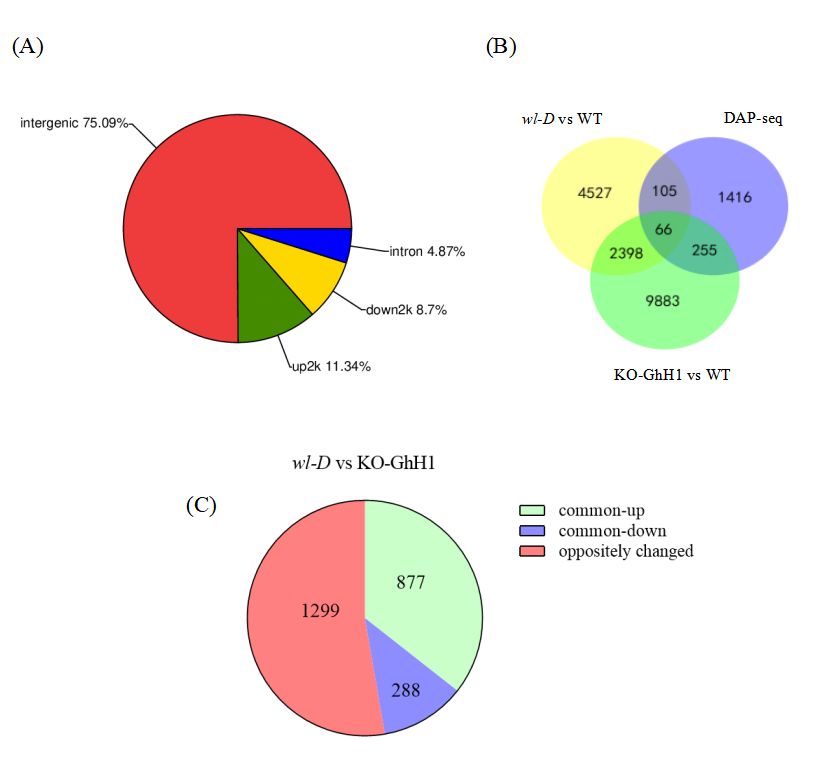


**Supplementary Figure 13.** (A) Locations of DAP-seq peaks across the cotton genome. (B) Venn diagram illustrating the overlap between differentially expressed genes (DEGs) in ***wl-D*** vs. WT and **KO*-GhH1*** vs. WT datasets with DAP-seq data. (C) Analysis of shared gene expression patterns.


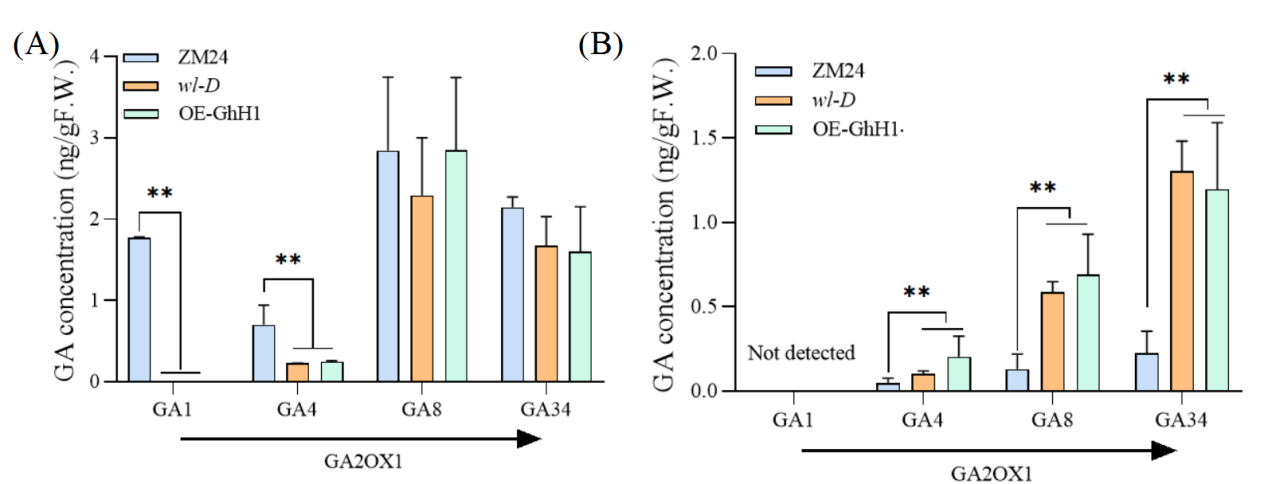


**Supplementary Figure 14. Quantification of Gibberellic Acid (GA) in ZM24, *wl-D*, and OE-*GhH1*.** (A) GA applied to juvenile leaves. (B) GA was applied to adult leaves. Data show mean ± SD (n = 3), ***P* < 0.01 (Student's *t-test*).


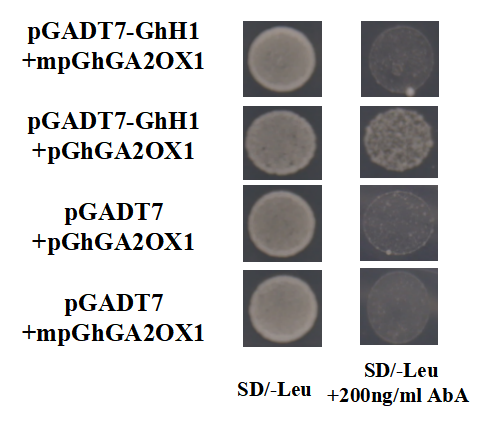


**Supplementary Figure 15.** **Y1H assay showing GhH1 binds to the *GhGA2OX1* promoter.** Binding occurred at the core sequence of ***GhGA2OX1***, but not at a mutated motif. SD/−Leu: SD medium without leucine; SD/−Leu/AbA_200_: SD medium without leucine and supplemented with 200 ng/mL AbA.


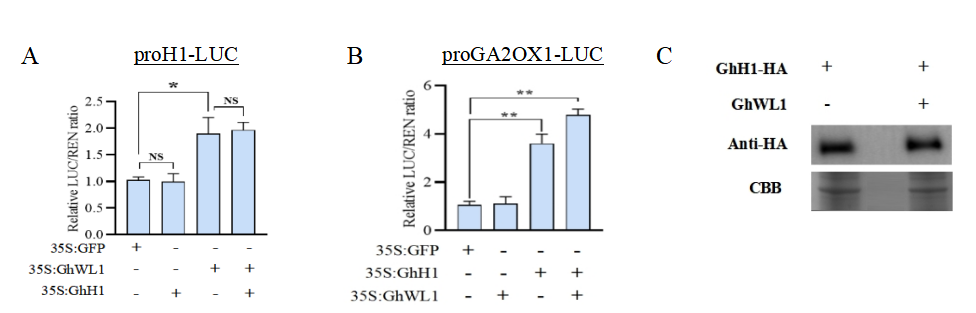


**Supplementary Figure 16.** Western blot analysis showing GhH1 protein expression in *Nicotiana benthamiana* leaves using an HA-tag antibody. Coomassie Brilliant Blue (CBB) staining served as a loading control.


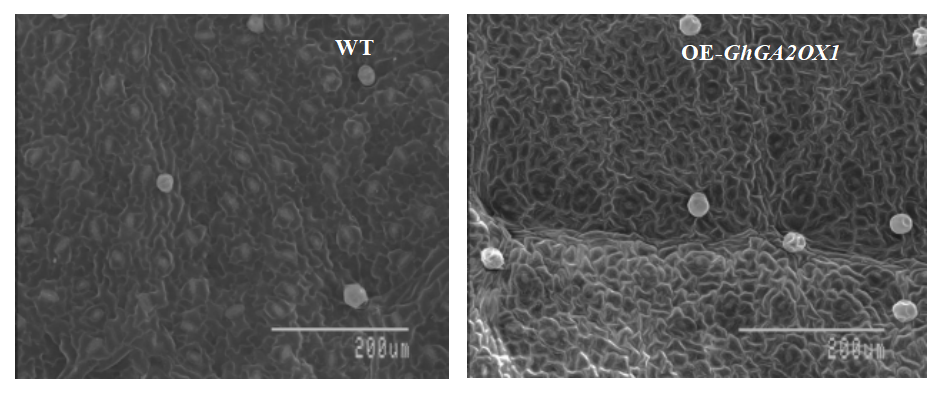


**Supplementary Figure 17.** **Scanning electron microscopy analysis (SEM) of leaf margins comparing mutant WT and OE-*GhGA2OX1*.**


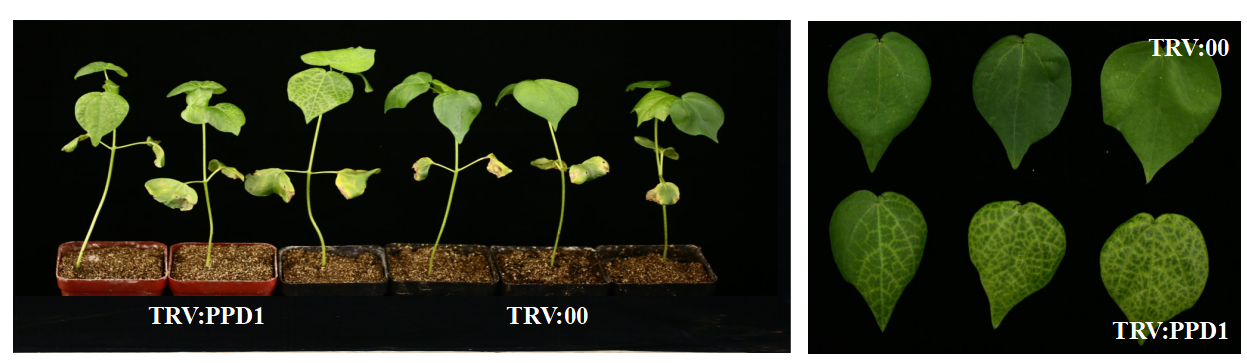
**Supplementary Figure 18. Phenotypic comparison of negative control and *GhPPD1*-silenced cotton plants.** TRV:00 represents the empty vector control, whereas TRV:GhPPD1 represents the cotton plants with silenced *GhPPD1*.


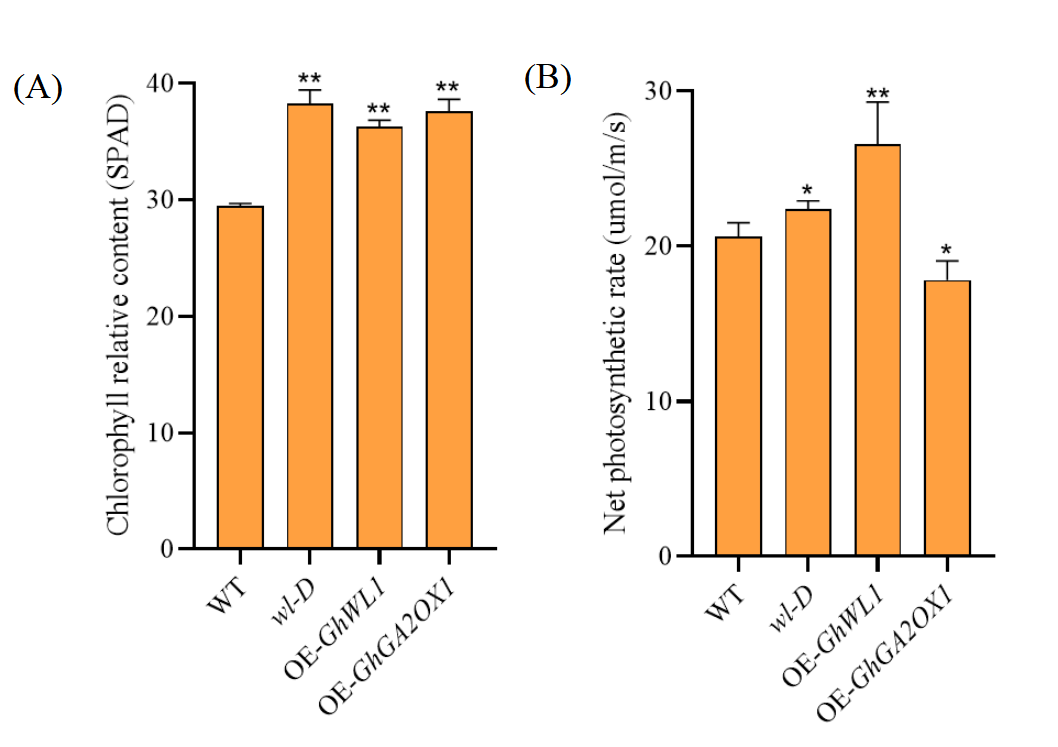


**Supplementary Figure 19. Leaf greenness index (A) and net photosynthetic rate (B) of WT, *wl-D*, OE-*GhWL1*, and OE-*GhGA2OX1*.** Data are presented as mean ± SD from three independent experiments (n > 3). **P* < 0.05, ***P* < 0.01 (Student's *t*-test).


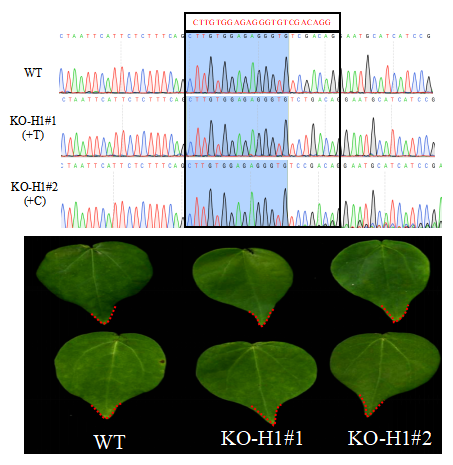


**Supplementary Figure 20. Leaf morphology of KO-*GhH1*.** Sanger sequencing analysis of WT (ZM24) and **KO-*GhH1*** mutants, illustrating insertion/deletion (InDel) patterns in CRISPR/Cas9-targeted ***GhH1*** mutants **(KO-*GhH1#1*** and **KO-*GhH1#2***). Inserted nucleotides (+N) are indicated.


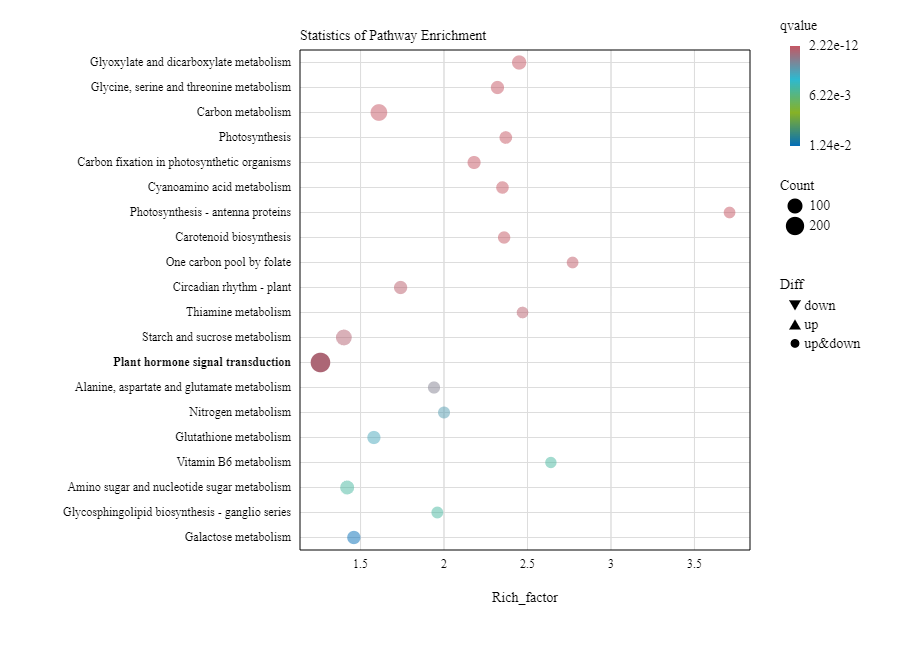


**Supplementary Figure 21.** **KEGG pathway enrichment analysis of differentially expressed genes (DEGs) between *wl-D* and ZM24.** The top 20 pathways are depicted, with the y-axis showing pathway names and the x-axis showing the rich factor. Circle size indicates the number of genes in each pathway.


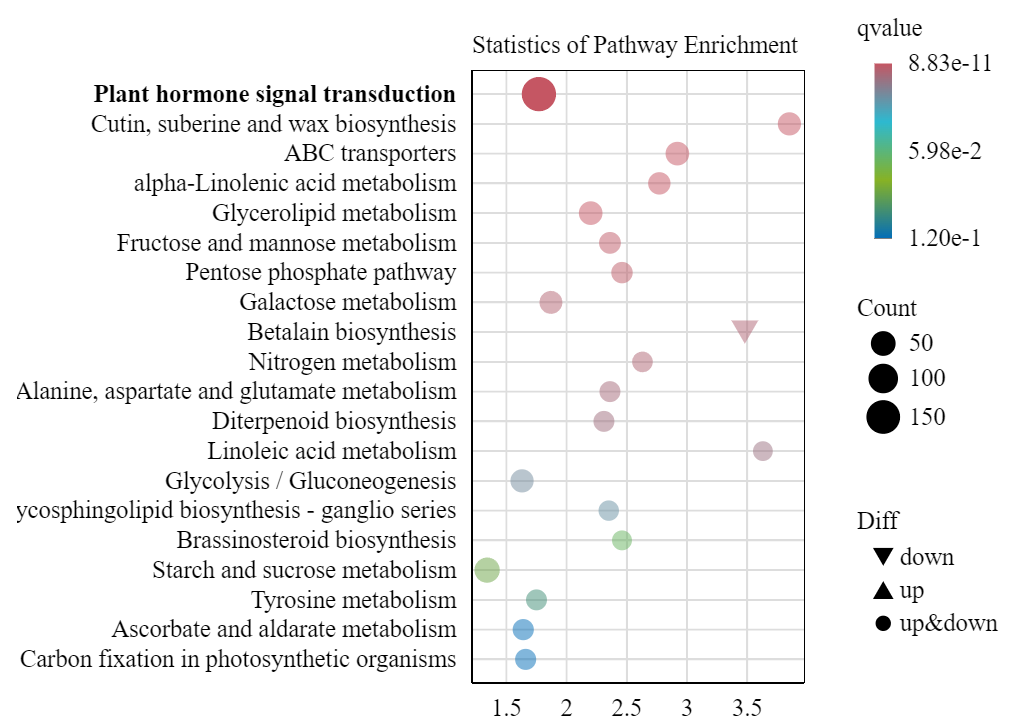


**Supplementary Figure 22.** **KEGG pathway enrichment analysis of DEGs between OE-*GhH1* and ZM24.** The top 20 pathways are depicted, with the y-axis showing pathway names and the x-axis showing the rich factor. Circle size indicates the number of genes in each pathway.
